# Anxiety or Depression Trends by Disability Status and Demographic Intersections in US Adults, 2019-2023

**DOI:** 10.1001/jamanetworkopen.2025.57332

**Published:** 2026-02-02

**Authors:** David Adzrago, Kayo Fujimoto, J. Michael Wilkerson, Typhanye V. Dyer, Faustine Williams

**Affiliations:** 1 Division of Intramural Research, National Institute on Minority Health and Health Disparities, National Institutes of Health, Bethesda, Maryland; 2 Department of Health Promotion and Behavioral Sciences, The University of Texas Health Science Center at Houston, Houston; 3 Department of Epidemiology and Biostatistics, University of Maryland at College Park, College Park

## Abstract

**Importance:**

Mental health problems, particularly anxiety and depression, remain among the leading causes of disease burden in the US. However, trends in anxiety and depression across disability status and demographics remain understudied.

**Objective:**

To examine trends in anxiety and depression prevalence among US adults from 2019 to 2023 by disability status and demographic characteristics.

**Design, Setting, and Participants:**

This cross-sectional study used national population-based data from the National Health Interview Surveys from 2019 to 2023. Data were collected from household probability samples of noninstitutionalized US civilian adults aged at least 18 years. Data analyses were performed from December 6, 2024, to November 19, 2025.

**Exposures:**

Disability status (measured using Washington Group on Disability Statistics Short Set on Functioning), race and ethnicity, sex, and nativity.

**Main Outcomes and Measures:**

The primary outcomes were self-reported anxiety or depression symptoms, assessed using the Washington Group on Disability Statistics Extended Set on Functioning. Joinpoint regression was used to estimate age-standardized prevalence trends and average annual percentage change (AAPC) across disability status, race and ethnicity, sex, and nativity.

**Results:**

A total of 150 220 adults were examined (81 525 [51.3%] female; 48 814 individuals [32.3%] aged 45-64 years; 126 051 individuals [81.1%] born in the US), including 16 172 Black individuals (11.9%), 20 427 Hispanic or Latino individuals (17.7%), 12 221 individuals (8.9%) who identified as another race or ethnicity, and 101 400 White individuals (61.4%). There were 15 519 respondents (8.2%) with general disability status and 60 248 respondents (42.3%) with anxiety or depression. From 2019 to 2023, anxiety or depression prevalence increased significantly among individuals without disabilities (AAPC, 3.93; 95% CI, 2.15-5.75). Prevalence increased across all racial and ethnic groups without disabilities, with the highest increases among Black respondents (AAPC, 4.77; 95% CI, 0.61-9.10) and respondents who identified as another race or ethnicity (AAPC, 6.95; 95% CI, 2.56-11.53). Prevalence increased only among females without disabilities (AAPC, 3.50; 95% CI, 2.14-4.87), while increases were observed among males with (AAPC, 3.25; 95% CI, 0.41-6.17) and without (AAPC, 4.62; 95% CI, 1.70-7.63) disabilities. Individuals born outside the US without disabilities experienced higher increases (AAPC, 5.57; 95% CI, 3.10-8.11) than those with disabilities (AAPC, 3.46; 95% CI, 0.06-6.98) and individuals born in the US without disabilities (AAPC, 3.75; 95% CI, 2.14-5.40). Across race and ethnicity, sex, and nativity intersections, Black female individuals born outside the US without disabilities exhibited the highest increase (AAPC, 14.89; 95% CI, 0.48-31.36).

**Conclusions and Relevance:**

This cross-sectional study found increasing trends in anxiety or depression prevalence, particularly among individuals without disabilities, individuals born outside the US, and Black individuals and those who did not identify as Black, Hispanic, or White, as well as males and individuals born outside the US with disabilities. These findings emphasize the need for targeted mental health interventions addressing disparities across disability status and demographic intersections.

## Key Points

**Question:**

Did anxiety and depression prevalence trends change over time from 2019 to 2023 among US adults across disability status and demographic characteristics?

**Findings:**

In this cross-sectional study of 150 220 adults, anxiety or depression prevalence increased significantly among individuals without disabilities, with differences by sex, nativity, and racial and ethnic groups. Among individuals with disabilities, prevalence increased in males and individuals born outside the US while Black females born outside the US without disabilities had the highest increase by race and ethnicity, sex, and nativity intersection.

**Meaning:**

These findings suggest the need for tailored mental health interventions that consider disability status and demographic intersections.

## Teaser

This cross-sectional study uses data from the National Health Interview Surveys to examine trends in the prevalence of anxiety or depression symptoms in US adults by disability status and demographic characteristics from 2019 to 2023.

## Introduction

Mental health problems, particularly anxiety and depression, the 2 most common and cooccurring mental health disorders,^1,2,3,4^ remain among the top 10 leading causes of disease and injury burdens in the US.^5^ Between 1990 and 2016, anxiety and depression accounted for approximately 31% and 27% increase, respectively, in years lived with disability.^5^ Anxiety and depression increased disability-adjusted life years (ie, the sum of years lived with anxiety- and depression-related disability and years of life lost due to anxiety- and depression-related premature death), by 16.7% and 17.3%, respectively, between 1990 and 2016.^5^ Thus, anxiety and depression disability-adjusted life years represent a health gap between a population’s current health state and a normative goal of living to an advanced age free of disease and disability.^5^

Trends in mental health problems across disability status, especially by intersection of demographic factors, such as race and ethnicity, sex, and nativity, remain understudied. Studies have reported higher mental health distress among US adults with disabilities, particularly among female and White individuals.^6,7^ However, how sex, race and ethnicity, and disability status intersect to influence mental health distress is less explored. US-born individuals have higher rates of mental health problems^8,9^ and disabilities^10,11^ than non–US-born individuals, yet the intersections of mental health, disability, and nativity have not been thoroughly investigated.

Evaluating and monitoring trends in mental health problems with respect to disability status and demographics are essential for identifying health disparities across populations, given the high burdens of mental health problems and disabilities. This population-based study assessed recent temporal trends in mental health problems among US adults by intersections of disability status, domains of disability, and demographics (race and ethnicity, sex, and nativity).

## Methods

### Data Source, Design, and Samples

This national serial cross-sectional study analyzed data on adults aged at least 18 years derived from 2019 through 2023 National Health Interview Surveys (NHISs). No institutional review board approval or informed consent were required for this study, as the datasets are deidentified and publicly available, in accordance with 45 CFR §46. We reported the results based on Strengthening the Reporting of Observational Studies in Epidemiology (STROBE) reporting guideline.

The NHIS is conducted yearly by the US Census Bureau among a nationally representative sample of noninstitutionalized US civilians living in the US.^12,13,14^ The NHIS methods are described in detail elsewhere.^14^ NHIS is a complex survey design with stratified cluster sampling in which households are systematically selected from counties within state boundaries, and individuals are randomly selected from each household.^12,13^ The response rates for the 2019 to 2023 surveys ranged from 47% in 2023 to 59% in 2019. The 2019 to 2023 surveys included 150 220 adults, with a larger sample size in 2019 (31 997 adults) and 2020 (31 568 adults) surveys than in 2023 (29 522 adults), 2021 (29 482 adults), and 2022 (27 651 adults) surveys. Of the total sample of 150 220 adults, 14 852 (9.89%) had missing values on anxiety or depression (3540 [2.36%]), general disability (20 [0.01%]), vision disability (81 [0.05%]), hearing disability (74 [0.05%]), mobility disability (64 [0.04%]), communication disability (51 [0.03%]), cognition disability (83 [0.06%]), self-care disability (53 [0.04%]), nativity (4884 [3.25%]), sex (16 [0.01%]), age (266 [0.24%]), and sexual orientation (5620 [3.74%]). There were no missing values on race and ethnicity and survey year. We used multiple imputations by chained equations with 20 datasets to impute missing values for the aforementioned variables.

### Measures

The primary outcome was self-reported anxiety and/or depression symptoms (hereinafter, *anxiety or depression symptoms*), which were assessed with 2 survey questions derived from the Washington Group on Disability Statistics Extended Set on Functioning. The first question asked participants how often they feel worried, nervous, or anxious. The second question asked them how often they feel depressed. The response options for each question include whether the participants experienced the symptoms daily, weekly, monthly, a few times a year, or never. Based on existing literature,^15,16^ we dichotomized the response options: participants had anxiety or depression symptoms if they reported daily, weekly, or monthly symptoms. Otherwise, participants did not have anxiety or depression symptoms if they reported the symptoms a few times a year or never had the symptoms. We estimated the prevalence of anxiety or depression by year, disability, race and ethnicity, sex, and nativity. Disability status was assessed based on 6 domains (vision, hearing, mobility, communication, cognition, and self-care) using Washington Group on Disability Statistics Short Set on Functioning.^14,17^ The questions asked about the participants’ level of difficulty (no difficulty, some difficulty, a lot of difficulty, or cannot do at all) in basic domains of functioning. Participants were classified with disability if they reported a lot of difficulty or cannot do at all for at least 1 of the 6 domains.^14,17^ Otherwise, the participants were classified with no disability if they reported some difficulty or no difficulty for at least 1 domain (and did not report a lot of difficulty or cannot do at all for any of the 6 domains).^17^ Race and ethnicity were self-reported and classified as Black, Hispanic or Latino, other (Alaska Native or Native American, Asian, and Native Hawaiian, or other single and multiple races), and White. Nativity was also self-reported by asking the participants whether they were born in the US (or US territory) or outside US.

### Statistical Analysis

We performed the data analyses from December 6, 2024, to November 19, 2025. Analyses were weighted using the NHIS weights to account for the complex survey design and to achieve nationally representative estimates of the noninstitutionalized civilian adult population.^14,17^ We used Stata statistical software version 18.0 (StataCorp) to calculate age-standardized anxiety/depression prevalence by survey year, disability status, race and ethnicity, sex, nativity, and disability domains. Age standardization was performed based on the direct method using ages 18 to 24 years, 25 to 34 years, 35 to 44 years, 45 to 64 years, and 65 years or older from the 2000 US Census population.^18^ We used Joinpoint regression software version 5.3.0 (National Cancer Institute)^19,20^ to calculate the trends and average annual percentage change (AAPC) in anxiety or depression prevalence, stratified by disability status, race and ethnicity, sex, nativity, and disability domains. The AAPC represents the summary measure of the trends in anxiety or depression prevalence from 2019 to 2023. The 95% CIs for the AAPCs were calculated using the parametric method, a preferred inference method in large sample size data analysis (including nationally representative data) compared with empirical quantile method, which is a nonparametric inference method, computationally more intensive, and generally preferred in smaller sample size and skewed data analysis.^21,22,23,24,25,26,27,28,29,30^ The permutation test with 4499 randomly permuted datasets was the model selection method, with the overall statistical significance set at a 2-sided *P* < .05.

## Results

### Population Characteristics

Of 150 220 adults included in the analysis, most were female (81 525 individuals [51.3%]) and born in the US (12 6051 individuals [81.1%]) (Table 1). There were 16 172 Black individuals (11.9%), 20 427 Hispanic or Latino individuals (17.7%), and 101 400 White individuals (61.4%). Of the total sample, 48 814 individuals (32.3%) were aged 45 to 64 years, 60 248 individuals (42.3%) experienced anxiety or depression, 15 519 individuals (8.2%) had general disability, 2514 individuals (1.4%) had vision disability, 2641 individuals (1.4%) had hearing disability, 9768 individuals (4.6%) had mobility disability, 1113 individuals (0.8%) had communication disability, 3733 individuals (2.5%) had cognition disability, and 1743 individuals (1.0%) had self-care disability.

**Table 1.** Sample Characteristics and Anxiety or Depression Prevalence Among US Adults

Characteristics	Respondents, No. (%) [95% CI]^a^		*P* value
	Overall sample (N = 150 220)	Anxiety or depression (n = 60 248)	
Anxiety/depression			
Yes	60 248 (42.3) [41.7-42.8]	NA	NA
No	89 972 (57.7) [57.2-58.3]	NA	
General disability status			
Yes	15 519 (8.2) [8.0-8.4]	8841 (68.8) [67.5-70.2]	<.001
No	134 701 (91.8) [91.6-92.0]	51 407 (40.0) [39.5-40.6]	
Vision disability			
Yes	2514 (1.4) [1.3-1.5]	1451 (64.7) [61.5-68.0]	<.001
No	147 706 (98.6) [98.5-98.7]	58 762 (42.0) [41.4-42.5]	
Hearing disability			
Yes	2641 (1.4) [1.3-1.5]	1391 (62.8) [58.9-66.7]	<.001
No	147 579 (98.6) [98.5-98.7]	58 816 (42.0) [41.4-42.5]	
Mobility disability			
Yes	9768 (4.6) [4.4-4.7]	5274 (65.4) [62.4-68.5]	<.001
No	140 452 (95.4) [95.3-95.6]	54 933 (41.1) [40.6-41.7]	
Communication disability			
Yes	1113 (0.8) [0.7-0.9]	677 (61.2) [56.6-65.8]	<.001
No	149 107 (99.2) [99.1-99.3]	59 523 (42.1) [41.6-42.7]	
Cognition disability			
Yes	3733 (2.5) [2.4-2.6]	2914 (81.9) [80.2-83.5]	<.001
No	146 487 (97.5) [97.4-97.6]	57 315 (41.3) [40.8-41.9]	
Self-care disability			
Yes	1743 (1.0) [0.9-1.1]	1109 (68.7) [64.5-72.8]	<.001
No	148 477 (99.0) [98.9-99.1]	59 103 (42.0) [41.4-42.5]	
Age, y			
18-24	9312 (11.6) [11.3-11.9]	5301 (55.2) [53.7-56.7]	<.001
25-34	21 902 (17.6) [17.3-17.9]	11 634 (51.4) [50.4-52.4]	
35-44	23 423 (16.6) [16.3-16.8]	10 804 (44.2) [43.2-45.1]	
45-64	48 814 (32.3) [31.9-32.6]	19 152 (37.7) [37.1-38.4]	
≥65	46 769 (21.9) [21.5-22.3]	13 357 (28.3) [27.8-28.9]	
Self-reported race and ethnicity			
Black	16 172 (11.9) [11.2-12.7]	5355 (34.4) [33.3-35.6]	<.001
Hispanic or Latino	20 427 (17.7) [16.5-19.0]	7390 (34.0) [33.1-34.9]	
Other single and multiple races^b^	12 221 (8.9) [8.3-9.6]	4171 (33.1) [32.0-34.2]	
White	101 400 (61.4) [60.0-62.8]	43 332 (48.3) [47.7-48.8]	
Sex			
Female	81 525 (51.3) [51.0-51.6]	36 892 (48.6) [47.9-49.3]	<.001
Male	68 695 (48.7) [48.4-49.0]	23 356 (35.7) [35.1-36.3]	
Nativity			
Born outside the US	24 169 (18.9] [18.0-19.8]	7097 (29.2) [28.3-30.0]	<.001
Born in the US	126 051 (81.1) [80.2-82.0]	53 151 (45.5) [44.9-46.0]	
Survey years			
2019	31 997 (19.8) [19.4-20.1]	12 113 (39.0) [38.2-39.8]	<.001
2020	31 568 (19.8) [19.5-20.2]	12 247 (40.5) [39.6-41.3]	
2021	29 482 (19.9) [19.6-20.3]	12 038 (42.5) [41.6-43.4]	
2022	27 651 (20.1) [19.7-20.5]	11 462 (44.7) [43.8-45.5]	
2023	29 522 (20.3) [19.8-20.9]	12 388 (44.7) [43.9-45.5]	

Abbreviation: NA, not applicable.

^a^ Data are from the National Health Interview Survey, 2019 to 2023. Frequencies are unweighted, and percentages are weighted.

^b^ Includes Alaska Native or Native American, Asian, and Native Hawaiian, or other single and multiple races. These categories were combined because of the small sample size and to increase statistical power.

### Prevalence of Anxiety or Depression According to Overall Disability Status and Demographics

Individuals with higher age-adjusted anxiety or depression prevalence were female, White, born in the US, and aged 18 to 24 years, had general disability or any specific disability (Table 1). For each year, the prevalence was significantly higher among individuals with general disability than among those without general disability (eTable 1 in Supplement 1). Overall, from 2019 to 2023, age-adjusted anxiety or depression prevalence was higher among people with disabilities—both overall and within specific disability domains and demographic groups—than those without disabilities (eFigures 1 to 29 in Supplement 1).

Across overall disability status, from 2019 to 2023, age-adjusted anxiety or depression prevalence increased significantly among individuals without disabilities annually (AAPC, 3.93; 95% CI, 2.15-5.75) (eTable 2 in Supplement 1). This significant upward trend was observed across all racial and ethnic groups without disabilities, with the highest increases among Black (AAPC, 4.77; 95% CI, 0.61-9.10) and other racial or ethnic groups (AAPC, 6.95; 95% CI, 2.56-11.53), compared with Hispanic or Latino (AAPC, 3.83; 95% CI, 1.66-6.04) and White (AAPC, 3.72; 95% CI, 1.85-5.63) groups (Table 2). While the prevalence increased only among females without disabilities (AAPC, 3.50; 95% CI, 2.14-4.87), it increased among males with (AAPC, 3.25; 95% CI, 0.41-6.17) and without (AAPC, 4.62; 95% CI, 1.70-7.63) disabilities, with a higher increase among males without disabilities (Table 3). The prevalence trends increased significantly for individuals born outside the US with (AAPC, 3.46; 95% CI, 0.06-6.98) and without (AAPC, 5.57; 95% CI, 3.10-8.11) disabilities, but increased only among US-born individuals without disabilities (AAPC, 3.75; 95% CI, 2.14-5.40) (Table 4). Individuals born outside the US had the highest prevalence increase.

**Table 2.** Trends in Anxiety or Depression by Race and Ethnicity and Disability Status Among US Adults, 2019-2023

Race and ethnicity and disability status	AAPC, % (95% CI)
Black	
No disability	4.77 (0.61 to 9.10)^a^
With disability	2.31 (−1.56 to 6.32)
Hispanic or Latino	
No disability	3.83 (1.66 to 6.04)^a^
With disability	3.05 (−0.69 to 6.94)
Other^b^	
No disability	6.95 (2.56 to 11.53)^a^
With disability	0.19 (−5.28 to 5.98)
White	
No disability	3.72 (1.85-5.63)^c^
With disability	1.92 (−2.31 to 6.33)

Abbreviation: AAPC, average annual percentage change.

^a^ Statistically significant at *P* < .05.

^b^ Includes Alaska Native or Native American, Asian, and Native Hawaiian, or other single and multiple races. These classifications or categories were combined due to the small sample size and to increase statistical power.

^c^ Statistically significant at *P* < .01.

**Table 3.** Trends in Anxiety or Depression by Sex and Disability Status Among US Adults, 2019-2023

Sex and disability status	AAPC, % (95% CI)
Female	
No disability	3.50 (2.14 to 4.87)^a^
With disability	1.02 (−1.42 to 3.53)
Male	
No disability	4.62 (1.70 to 7.63)^b^
With disability	3.25 (0.41 to 6.17)^b^

Abbreviation: AAPC, average annual percentage change.

^a^ Statistically significance at *P* < .01.

^b^ Statistically significance at *P* < .05.

**Table 4.** Trends in Anxiety or Depression by Nativity and Disability Status Among US Adults, 2019-2023

Nativity and disability status	AAPC, % (95% CI)
Born outside the US	
No disability	5.57 (3.10 to 8.11)^a^
With disability	3.46 (0.06 to 6.98)^b^
Born in the US	
No disability	3.75 (2.14 to 5.40)^a^
With disability	1.92 (−1.11 to 5.05)

Abbreviation: AAPC, average annual percentage change.

^a^ Statistically significant at *P* < .01.

^b^ Statistically significant at *P* < .05.

When we examined the prevalence changes by the intersection of race and ethnicity, sex, nativity, and disability status (Table 5), Black females born outside the US without disabilities experienced the most significant increases. Notably, the prevalence trends increased for Black females born outside the US (AAPC, 14.89; 95% CI, 0.48-31.36) and Black males born outside the US (AAPC, 8.26; 95% CI, 3.55-13.18) without disabilities. The upward trends were similar among Hispanic or Latino males without disabilities who were born outside the US (AAPC, 5.31; 95% CI, 3.43-7.22) and born in the US (AAPC, 5.06; 95% CI, 2.26-7.95). The increased prevalence was higher among White males born outside the US (AAPC, 7.79; 95% CI, 1.64-14.32) than among White males born in the US (AAPC, 4.41; 95% CI, 1.02-7.92) and White females born in the US (AAPC, 3.45; 95% CI, 2.08-4.82) without disabilities. However, there were no statistically significant annual prevalence changes among persons with disabilities, regardless of race and ethnicity or the intersection of race and ethnicity, sex, and nativity.

**Table 5.** Trends in Anxiety or Depression by Race and Ethnicity, Sex, Nativity, and Disability Status Among US Adults, 2019-2023

Race and/or ethnicity, sex, nativity, and disability status	AAPC, % (95% CI)
**Black females**	
Born outside the US	
No disability	14.89 (0.48 to 31.36)^a^
With disability	1.71 (−32.07 to 52.27)
Born in the US	
No disability	4.57 (−0.41 to 9.80)
With disability	1.79 (−0.60 to 4.24)
**Black males**	
Born outside the US	
No disability	8.26 (3.55 to −13.18)^a^
With disability	5.14 (−15.03 to 30.09)
Born in the US	
No disability	4.16 (−0.99 to 9.57)
With disability	2.43 (−7.09 to 12.92)
**Hispanic females**	
Born outside the US	
No disability	3.87 (−1.39 to 9.41)
With disability	−1.34 (−11.87 to 10.45)
Born in the US	
No disability	1.26 (−2.61 to 5.27)
With disability	0.45 (−11.27 to 13.71)
**Hispanic males**	
Born outside the US	
No disability	5.31 (3.43 to 7.22)^b^
With disability	7.91 (−9.94 to 29.29)
Born in the US	
No disability	5.06 (2.26 to 7.95)^a^
With disability	3.50 (−3.34 to 10.83)
**Other females** ^c^	
Born outside the US	
No disability	8.46 (−1.12 to 18.97)
With disability	6.00 (−18.57 to 37.98)
Born in the US	
No disability	3.09 (−0.56 to 6.87)
With disability	−0.31 (−7.99 to 8.00)
**Other males** ^c^	
Born outside the US	
No disability	10.68 (−4.39 to 28.12)
With disability	−7.75 (−36.79 to 34.63)
Born in the US	
No disability	4.53 (−8.40 to 19.29)
With disability	0.31 (−5.67 to 6.67)
**White females**	
Born outside the US	
No disability	−1.07 (−4.64 to 2.63)
With disability	−4.94 (−13.95 to 5.01)
Born in the US	
No disability	3.45 (2.08 to 4.82)^b^
With disability	1.66 (−1.38 to −4.80)
**White males**	
Born outside the US	
No disability	7.79 (1.64 to 14.32)^a^
With disability	3.66 (−8.93 to 17.99)
Born in the US	
No disability	4.41 (1.02 to 7.92)^a^
With disability	2.76 (−2.58 to 8.39)

Abbreviation: AAPC, average annual percentage change.

^a^ Statistical significant at *P* < .05.

^b^ Statistical significant at *P* < .01.

^c^ Includes Alaska Native or Native American, Asian, and Native Hawaiian, or other single and multiple races. These classifications or categories were combined due to the small sample size and to increase statistical power.

### Prevalence of Anxiety or Depression According to Disability Domains and Demographics

From 2019 to 2023, the average annual age-adjusted anxiety or depression prevalence increases were relatively similar across the 6 disability domains (vision, hearing, mobility, communication, cognition, and self-care) (eTables 3, 7, 11, 15, 19, and 23 in Supplement 1). Across the 6 disability domains and race and ethnicity, sex, and nativity, average annual prevalences increased significantly among individuals without specific disability, especially among racial and ethnic minority groups (ie, Black, Hispanic or Latino, and others), males, and individuals born outside the US (eTables 4-6, 8-10, 12-14, 16-18, 20-22, and 24-26 in Supplement 1). Among people with vision, hearing, mobility, cognition, or self-care disability, the AAPCs were not statistically significant across any of the demographic factors. However, the changes were significant and unique across sex among people with or without communication disability; while the average annual prevalence decreased for males with communication disability (AAPC, −3.57; 95% CI, −5.98 to −1.10), it increased for males (AAPC, 4.64; 95% CI, 1.72-7.65) and females (AAPC, 3.24; 95% CI, 2.05-4.44) without communication disability (eTable 17 in Supplement 1).

## Discussion

In this population-based cross-sectional study, we identified distinct trends of age-adjusted anxiety or depression symptom prevalence by disability status, disability domains, race and ethnicity, sex, and nativity among US adults. We found similar increasing prevalence trends across all 6 disability domains and the overall disability status, particularly among people without disabilities. Notably, prevalence increased among persons with disabilities identifying as male or born outside the US. Increased prevalence was also observed among persons without disabilities across sex, nativity, or race and ethnicity. At the intersections of race and ethnicity, sex, and nativity, we found increasing trends among Black females and males born outside the US, Hispanic or Latino males (regardless of nativity), and White males and females (regardless of nativity) without disabilities. These findings emphasize the need to consider and monitor disability status–related differences in anxiety and depression trends across demographics for personalized programs to address mental health disparities in the US.

The prevalence of anxiety or depression was higher among persons with disabilities than those without disabilities across each year, which is consistent with previous studies.^6,7,31^ Despite the protections (eg, adequate housing and accessible public spaces) and programs (ie, equal access to state and local government programs) established by the Americans with Disabilities Act (ADA),^32,33,34^ mental health disparities among people with disabilities persist. People with disabilities continue to face greater barriers to health care and limited social supports, increasing their risk for poor mental health and unmet care needs.^35,36,37,38^ However, the average annual prevalence increases between 2019 and 2023 were greater among individuals without disabilities than those with disabilities across the 6 disability domains (vision, hearing, mobility, communication, cognition, and self-care). Nonetheless, these increases were not statistically significant among the population with general disabilities or within specific disability domains. These patterns reflect gaps in mental health and disability programs that require further attention.^34,35,39,40^ People with disabilities have higher mental health problems and experience more socioeconomic disadvantages (eg, stigma, prejudice, employment, education, transportation, and internet access issues) than those without disabilities.^35^ The lack of significant average annual changes in prevalence among persons with disabilities, coupled with significant increases among persons without disabilities, necessitate further exploration to expand the literature.

Some of the patterns of anxiety or depression prevalence across disability status might be explained by social determinants of health, including sociodemographic characteristics, that influence population well-being and disparities.^41,42^ Consistent with previous studies,^6,7^ we found a higher anxiety or depression prevalence among persons with disabilities than those without disabilities across each year and all racial and ethnic groups, sex, and nativity groups. Notably, we observed a higher prevalence among White individuals, females, and individuals born in the US with disabilities. Within this subgroup, only males and individuals born outside the US experienced increased average annual prevalence between 2019 and 2023, suggesting unique gaps in mental health and disability services. Similarly, significantly increased trends were also observed among persons without disabilities who identified as female, male, born outside the US, born in the US, Black, Hispanic or Latino, White, or other racial or ethnic group.

Mental health disparities were also pronounced when examined by the intersections of race and ethnicity, sex, nativity, and disability status among persons without disabilities. Particularly, individuals with multiple intersecting identities (eg, Black females and males born outside the US, Hispanic males born in or outside the US, White males born outside the US, and White males and females born in the US) exhibited significantly increasing trends in average annual anxiety or depression prevalence among people without disabilities. As noted in previous studies,^42,43,44^ the rising trends and disparities in anxiety or depression may be due to lack of consideration of multiple intersecting social determinants of health in mental health research and interventions, as well as limited mental health programs. Also, the lack of significant changes in the average prevalence among individuals with disabilities suggests inadequate improvements in mental health and disability programs emphasizing the need for targeted program improvements.^35^

### Limitations

This study has limitations. First, all measures were self-reported and therefore susceptible to recall and social desirability biases, which could have led to underreporting. Second, the NHIS only surveys the civilian noninstitutionalized US population, excluding those living in long-term care or residential facilities. Third, because NHIS is administered only in English, our findings may not be generalizable to the entire US adult population. Fourth, we did not estimate the APC to characterize trends in anxiety or depression prevalence over time because we had fewer than the 7 data point minimum required to allow a joinpoint.^19,45^

## Conclusions

In this national serial cross-sectional study, we found notable variations in mental health problems based on disability status and specific social determinants of health among adults. The consistently high prevalence of anxiety or depression symptoms among individuals with disabilities emphasizes the need for improvements in disability and mental health programs and policies. The increasing trends across disability status and domains varied by race and ethnicity, sex, and nativity, as well as the intersection of these factors. These findings underscore the importance of social determinants of health, particularly their multiplicative effects, in delineating disparities in mental health and disabilities. Expanding evidence-based campaigns and demographically tailored mental health services is necessary to reduce the widening and persistent health disparities.

## Notes

Supplement 1. eFigure 1. Trends in Age-Standardized Anxiety/Depression Prevalence by Disability Status Among Adults in the United States, 2019–2023 eFigure 2. Trends in Age-Standardized Anxiety/Depression Prevalence by Disability Status and Race and/or Ethnicity Among Adults in the United States, 2019–2023 eFigure 3. Trends in Age-Standardized Anxiety/Depression Prevalence by Disability Status and Sex Among Adults in the United States, 2019–2023 eFigure 4. Trends in Age-Standardized Anxiety/Depression Prevalence by Disability Status and Nativity Among Adults in the United States, 2019–2023 eFigure 5. Trends in Age-Standardized Anxiety/Depression Prevalence by Disability Status, Race and/or Ethnicity, Sex, and Nativity Among Adults in the United States, 2019–2023 eFigure 6. Trends in Age-Standardized Anxiety/Depression Prevalence by Vision Disability Status Among Adults in the United States, 2019–2023 eFigure 7. Trends in Age-Standardized Anxiety/Depression Prevalence by Vision Disability Status and Race and/or Ethnicity Among Adults in the United States, 2019–2023 eFigure 8. Trends in Age-Standardized Anxiety/Depression Prevalence by Vision Disability Status and Sex Among Adults in the United States, 2019–2023 eFigure 9. Trends in Age-Standardized Anxiety/Depression Prevalence by Vision Disability Status and Nativity Among Adults in the United States, 2019–2023 eFigure 10. Trends in Age-Standardized Anxiety/Depression Prevalence by Hearing Disability Status Among Adults in the United States, 2019–2023 eFigure 11. Trends in Age-Standardized Anxiety/Depression Prevalence by Hearing Disability Status and Race and/or Ethnicity Among Adults in the United States, 2019–2023 eFigure 12. Trends in Age-Standardized Anxiety/Depression Prevalence by Hearing Disability Status and Sex Among Adults in the United States, 2019–2023 eFigure 13. Trends in Age-Standardized Anxiety/Depression Prevalence by Hearing Disability Status and Nativity Among Adults in the United States, 2019–2023 eFigure 14. Trends in Age-Standardized Anxiety/Depression Prevalence by Mobility Disability Status Among Adults in the United States, 2019–2023 eFigure 15. Trends in Age-Standardized Anxiety/Depression Prevalence by Mobility Disability Status and Race and/or Ethnicity Among Adults in the United States, 2019–2023 eFigure 16. Trends in Age-Standardized Anxiety/Depression Prevalence by Mobility Disability Status and Sex Among Adults in the United States, 2019–2023 eFigure 17. Trends in Age-Standardized Anxiety/Depression Prevalence by Mobility Disability Status and Nativity Among Adults in the United States, 2019–2023 eFigure 18. Trends in Age-Standardized Anxiety/Depression Prevalence by Communication Disability Status Among Adults in the United States, 2019–2023 eFigure 19. Trends in Age-Standardized Anxiety/Depression Prevalence by Communication Disability Status and Race and/or Ethnicity Among Adults in the United States, 2019–2023 eFigure 20. Trends in Age-Standardized Anxiety/Depression Prevalence by Communication Disability Status and Sex Among Adults in the United States, 2019–2023 eFigure 21. Trends in Age-Standardized Anxiety/Depression Prevalence by Communication Disability Status and Nativity Among Adults in the United States, 2019–2023 eFigure 22. Trends in Age-Standardized Anxiety/Depression Prevalence by Cognition Disability Status Among Adults in the United States, 2019–2023 eFigure 23. Trends in Age-Standardized Anxiety/Depression Prevalence by Cognition Disability Status and Race and/or Ethnicity Among Adults in the United States, 2019–2023 eFigure 24. Trends in Age-Standardized Anxiety/Depression Prevalence by Cognition Disability Status and Sex Among Adults in the United States, 2019–2023 eFigure 25. Trends in Age-Standardized Anxiety/Depression Prevalence by Cognition Disability Status and Nativity Among Adults in the United States, 2019–2023 eFigure 26. Trends in Age-Standardized Anxiety/Depression Prevalence by Self-care Disability Status Among Adults in the United States, 2019–2023 eFigure 27. Trends in Age-Standardized Anxiety/Depression Prevalence by Self-care Disability Status and Race and/or Ethnicity Among Adults in the United States, 2019–2023 eFigure 28. Trends in Age-Standardized Anxiety/Depression Prevalence by Self-care Disability Status and Sex Among Adults in the United States, 2019–2023 eFigure 29. Trends in Age-Standardized Anxiety/Depression Prevalence by Self-care Disability Status and Nativity Among Adults in the United States, 2019–2023 eTable 1. Anxiety/Depression Prevalence by Disability Status and Survey Year Among Adults in the United States eTable 2. Trends in Anxiety/Depression by Disability Status Among Adults in the United States, 2019–2023 eTable 3. Trends in Anxiety/Depression by Vision Disability Status Among Adults in the United States, 2019–2023 eTable 4. Trends in Anxiety/Depression by Race and/or Ethnicity and Vision Disability Status Among Adults in the United States, 2019–2023 eTable 5. Trends in Anxiety/Depression by Sex and Vision Disability Status Among Adults in the United States, 2019–2023 eTable 6. Trends in Anxiety/Depression by Nativity and Vision Disability Status Among Adults in the United States, 2019–2023 eTable 7. Trends in Anxiety/Depression by Hearing Disability Status Among Adults in the United States, 2019–2023 eTable 8. Trends in Anxiety/Depression by Race and/or Ethnicity and Hearing Disability Status Among Adults in the United States, 2019–2023 eTable 9. Trends in Anxiety/Depression by Sex and Hearing Disability Status Among Adults in the United States, 2019–2023 eTable 10. Trends in Anxiety/Depression by Nativity and Hearing Disability Status Among Adults in the United States, 2019–2023 eTable 11. Trends in Anxiety/Depression by Mobility Disability Status Among Adults in the United States, 2019–2023 eTable 12. Trends in Anxiety/Depression by Race and/or Ethnicity and Mobility Disability Status Among Adults in the United States, 2019–2023 eTable 13. Trends in Anxiety/Depression by Sex and Mobility Disability Status Among Adults in the United States, 2019–2023 eTable 14. Trends in Anxiety/Depression by Nativity and Mobility Disability Status Among Adults in the United States, 2019–2023 eTable 15. Trends in Anxiety/Depression by Communication Disability Status Among Adults in the United States, 2019–2023 eTable 16. Trends in Anxiety/Depression by Race and/or Ethnicity and Communication Disability Status Among Adults in the United States, 2019–2023 eTable 17. Trends in Anxiety/Depression by Sex and Communication Disability Status Among Adults in the United States, 2019–2023 eTable 18. Trends in Anxiety/Depression by Nativity and Communication Disability Status Among Adults in the United States, 2019–2023 eTable 19. Trends in Anxiety/Depression by Cognition Disability Status Among Adults in the United States, 2019–2023 eTable 20. Trends in Anxiety/Depression by Race and/or Ethnicity and Cognition Disability Status Among Adults in the United States, 2019–2023 eTable 21. Trends in Anxiety/Depression by Sex and Cognition Disability Status Among Adults in the United States, 2019–2023 eTable 22. Trends in Anxiety/Depression by Nativity and Cognition Disability Status Among Adults in the United States, 2019–2023 eTable 23. Trends in Anxiety/Depression by Self-care Disability Status Among Adults in the United States, 2019–2023 eTable 24. Trends in Anxiety/Depression by Race and/or Ethnicity and Self-care Disability Status Among Adults in the United States, 2019–2023 eTable 25. Trends in Anxiety/Depression by Sex and Self-care Disability Status Among Adults in the United States, 2019–2023 eTable 26. Trends in Anxiety/Depression by Nativity and Self-care Disability Status Among Adults in the United States, 2019–2023

Supplement 2. Data Sharing Statement

## References

[1] World Health Organization. Depression and other common mental disorders: global health estimates. Accessed March 11, 2025. https://iris.who.int/bitstream/handle/10665/254610/WHO-MSD-MER-2017.2-eng.pdf?sequence=1

[2] Vos T, Allen C, Arora M, ; GBD 2015 Disease and Injury Incidence and Prevalence Collaborators. Global, regional, and national incidence, prevalence, and years lived with disability for 310 diseases and injuries, 1990-2015: a systematic analysis for the Global Burden of Disease Study 2015. Lancet. 2016;388(10053):1545-1602. doi:10.1016/S0140-6736(16)31678-6 27733282 PMC5055577

[3] Kalin NH. The Critical Relationship Between Anxiety and Depression. American Psychiatric Association; 2020:365-367. 10.1176/appi.ajp.2020.20030305 32354270

[4] Anxiety & Depression Association of America. Anxiety disorders—facts & statistics. Accessed March 11, 2025. https://adaa.org/understanding-anxiety/facts-statistics

[5] Mokdad AH, Ballestros K, Echko M, ; US Burden of Disease Collaborators. The state of US health, 1990-2016: burden of diseases, injuries, and risk factors among US states. JAMA. 2018;319(14):1444-1472. doi:10.1001/jama.2018.0158 29634829 PMC5933332

[6] Czeisler MÉBA, Board A, Thierry JM, . Mental health and substance use among adults with disabilities during the COVID-19 pandemic—United States, February–March 2021. MMWR Morb Mortal Wkly Rep. 2021;70(34):1142-1149. doi:10.15585/mmwr.mm7034a3 34437518 PMC8389385

[7] Cree RAOC, Okoro CA, Zack MM, Carbone E. Frequent mental distress among adults, by disability status, disability type, and selected characteristics—United States, 2018. MMWR Morb Mortal Wkly Rep. 2020;69(36):1238-1243. doi:10.15585/mmwr.mm6936a2 32914770 PMC7499832

[8] Dedania R, Gonzales G. Disparities in access to health care among US-born and foreign-born US adults by mental health status, 2013-2016. Am J Public Health. 2019;109(S3):S221-S227. doi:10.2105/AJPH.2019.305149 31242018 PMC6595516

[9] Salas-Wright CP, Vaughn MG, Goings TC, Miller DP, Schwartz SJ. Immigrants and mental disorders in the united states: new evidence on the healthy migrant hypothesis. Psychiatry Res. 2018;267:438-445. doi:10.1016/j.psychres.2018.06.039 29980122 PMC6131041

[10] Burns SD, Baker EH, Sheehan CM. Disability and self-rated health: exploring foreign- and U.S.-born differences across adulthood. J Migr Health. 2022;6:100112. doi:10.1016/j.jmh.2022.100112 35620793 PMC9126965

[11] Zajacova A, Margolis R. Trends in disability and limitations among US adults aged 18-44 years, 2000-2018. Am J Epidemiol. 2025;194(5):1381-1388. doi:10.1093/aje/kwae262 39136389 PMC12055464

[12] Keralis JM, Zhang C, Cox CS, Mirel LB. A comparison of the mortality experience of U.S. adults estimated with the 2006-2018 National Health Interview Survey linked mortality files and the annual U.S. Life Tables. Natl Health Stat Report. 2023;(186):1-29. 37252817

[13] Moriarity C, Parsons VL, Jonas K, Schar BG, Bose J, Bramlett MD. Sample design and estimation structures for the National Health Interview Survey, 2016-2025. Vital Health Stat 1. 2022;(191):1-30. doi:10.15620/cdc:115394 35796667

[14] National Center for Health Statistics. NHIS questionnaires, datasets, and documentation. Accessed December 17, 2025. https://www.cdc.gov/nchs/nhis/documentation/?CDC_AAref_Val=https://www.cdc.gov/nchs/nhis/data-questionnaires-documentation.htm

[15] Zablotsky B, Weeks JD, Terlizzi EP, Madans JH, Blumberg SJ. Assessing anxiety and depression: a comparison of national health interview survey measures. Natl Health Stat Report. 2022;(172):1-17. doi:10.15620/cdc:117491 35876842

[16] Adzrago D, Thapa K, Rajbhandari-Thapa J, Sulley S, Williams F. Influence of biopsychosocial factors on self-reported anxiety/depression symptoms among first-generation immigrant population in the U.S. BMC Public Health. 2024;24(1):819. doi:10.1186/s12889-024-18336-w 38491362 PMC10941619

[17] Weeks JD, Dahlhamer JM, Madans JH, Maitland A. Measuring disability: an examination of differences between the Washington Group Short Set on Functioning and the American Community Survey disability questions. Natl Health Stat Report. 2021;(161):1-9. doi:10.15620/cdc:107202 34546873

[18] Klein RJ, Schoenborn CA. Age adjustment using the 2000 projected U.S. population. Healthy People 2010 Stat Notes. 2001;(20):1-10. 11676466

[19] Irimata KE, Bastian BA, Clarke TC, Curtin SC, Badwe R, Rui P. Guidance for Selecting Model Options in the National Cancer Institute Joinpoint Regression Software. Vital Health Stat 1. 2022;(194):1-22. doi:10.15620/cdc:118050 36255743

[20] National Cancer Institute. Joinpoint. Accessed July 1, 2024. https://surveillance.cancer.gov/help/joinpoint

[21] Asmare E, Begashaw A. Review on parametric and nonparametric methods of efficiency analysis. Biostat Bioinform. 2018;2(2):1-7. doi:10.31031/OABB.2018.02.000534

[22] Barber CA, Chien LC, Labus B, . Application of Joinpoint regression to SARS-CoV-2 wastewater-based epidemiology in Las Vegas, Nevada, USA. Epidemiol Infect. 2025;153:e68. doi:10.1017/S0950268825100058 40488402 PMC12171900

[23] Eikermann GM, Ludeke CM, Eyth A, . Racial/ethnic and regional disparities in opioid-involved overdose deaths among children and adolescents in the United States. J Racial Ethn Health Disparities. Published online July 24, 2025. doi:10.1007/s40615-025-02554-y 40707842 PMC13546327

[24] Irimata KE, Malec DJ, Bastian BA, Spencer MR. Using SAS/STAT to understand the NCI Joinpoint regression software: testing for a zero slope using rates of drugs overdose deaths involving fentanyl, 2011–2016. Natl Health Stat Rep. 2021;(156):1-15. doi:10.15620/cdc:105105 34181517

[25] Li X, Qu P, Yan P, . Trends in burden and mortality of congenital birth defects in G20 countries (1990-2021) and predictions for 2022-2040. BMC Pregnancy Childbirth. 2025;25(1):494. doi:10.1186/s12884-025-07617-w 40281466 PMC12023674

[26] Liu B, Kim HJ, Feuer EJ, Graubard BI. Joinpoint regression methods of aggregate outcomes for complex survey data. J Surv Stat Methodol. 2023;11(4):967-989. doi:10.1093/jssam/smac014

[27] Rana RK, Singhal R, Dua P. Deciphering the dilemma of parametric and nonparametric tests. J Pract Cardiovasc Sci. 2016;2(2):95-98. doi:10.4103/2395-5414.191521

[28] Uttley J. Power analysis, sample size, and assessment of statistical assumptions—improving the evidential value of lighting research. Leukos. 2019;15(2-3):143-162. doi:10.1080/15502724.2018.1533851

[29] Vrbin CM. Parametric or nonparametric statistical tests: Considerations when choosing the most appropriate option for your data. Cytopathology. 2022;33(6):663-667. doi:10.1111/cyt.13174 36017662

[30] Xu S, Murtagh S, Han Y, Wan F, Toriola AT. Breast cancer incidence among US women aged 20 to 49 years by race, stage, and hormone receptor status. JAMA Netw Open. 2024;7(1):e2353331. doi:10.1001/jamanetworkopen.2023.53331 38277147 PMC10818222

[31] Koenig J, McLean KJ, Bishop L. Psychological distress and mental health diagnoses in adults by disability and functional difficulty status: findings from the 2021 national health interview survey. Disabil Health J. 2024;17(4):101641. doi:10.1016/j.dhjo.2024.101641 38816306 PMC11401770

[32] Harris SP, Gould R, Mullin C. ADA research brief: Mental health, employment and the ADA. Accessed March 5, 2025. https://adata.org/research_brief/mental-health-employment-and-ada

[33] Blanck P. On the importance of the Americans with Disabilities Act at 30. J Disabil Policy Stud. 2023;2023(34):176-198. doi:10.1177/10442073211036900 39286446 PMC11404550

[34] Burnim I. The promise of the Americans with Disabilities Act for people with mental illness. JAMA. 2015;313(22):2223-2224. doi:10.1001/jama.2015.4015 26057279

[35] Krahn GL, Walker DK, Correa-De-Araujo R. Persons with disabilities as an unrecognized health disparity population. Am J Public Health. 2015;105(Suppl 2)(suppl 2):S198-S206. doi:10.2105/AJPH.2014.302182 25689212 PMC4355692

[36] Okoro CA, Hollis ND, Cyrus AC, Griffin-Blake S. Prevalence of disabilities and health care access by disability status and type among adults—United States, 2016. MMWR Morb Mortal Wkly Rep. 2018;67(32):882-887. doi:10.15585/mmwr.mm6732a3 30114005 PMC6095650

[37] de la Vega R, Molton IR, Miró J, Smith AE, Jensen MP. Changes in perceived social support predict changes in depressive symptoms in adults with physical disability. Disabil Health J. 2019;12(2):214-219. doi:10.1016/j.dhjo.2018.09.005 30314820

[38] Tough H, Siegrist J, Fekete C. Social relationships, mental health and wellbeing in physical disability: a systematic review. BMC Public Health. 2017;17(1):414. doi:10.1186/s12889-017-4308-6 28482878 PMC5422915

[39] Mongelli F, Georgakopoulos P, Pato MT. Challenges and opportunities to meet the mental health needs of underserved and disenfranchised populations in the United States. Focus (Am Psychiatr Publ). 2020;18(1):16-24. doi:10.1176/appi.focus.20190028 32047393 PMC7011222

[40] Pollack HA, Berg KL. Meeting the mental health needs of Americans who live with intellectual and developmental disabilities. *Milbank Quarterly Opinion*. March 25, 2024. Accessed December 17, 2025. https://www.milbank.org/quarterly/opinions/meeting-the-mental-health-needs-of-americans-who-live-with-intellectual-and-developmental-disabilities/

[41] Singh GK, Daus GP, Allender M, . Social determinants of health in the United States: addressing major health inequality trends for the nation, 1935-2016. Int J MCH AIDS. 2017;6(2):139-164. doi:10.21106/ijma.236 29367890 PMC5777389

[42] Kirkbride JB, Anglin DM, Colman I, . The social determinants of mental health and disorder: evidence, prevention and recommendations. World Psychiatry. 2024;23(1):58-90. doi:10.1002/wps.21160 38214615 PMC10786006

[43] McGINTY B. The future of public mental health: challenges and opportunities. Milbank Q. 2023;101(S1)(suppl 1):532-551. doi:10.1111/1468-0009.12622 37096616 PMC10126977

[44] Barksdale CL, Pérez-Stable E, Gordon J. Innovative directions to advance mental health disparities research. Am J Psychiatry. 2022;179(6):397-401. doi:10.1176/appi.ajp.21100972 35599539

[45] Ingram DD, Malec DJ, Makuc DM, . National Center for Health Statistics guidelines for analysis of trends. Vital Health Stat 2. 2018;(179):1-71. 29775435

