# Supplementary material for: Anxiety or Depression Trends by Disability Status and Demographic Intersections in US Adults, 2019-2023
Source: JAMA Netw Open. 2026 Feb 2;9(2):e2557332. doi: 10.1001/jamanetworkopen.2025.57332 (PMC12865658; doi:10.1001/jamanetworkopen.2025.57332)
Supplement: Supplement 1. — eFigure 1. Trends in Age-Standardized Anxiety/Depression Prevalence by Disability Status Among Adults in the United States, 2019–2023 eFigure 2. Trends in Age-Standardized Anxiety/Depression Prevalence by Disability Status and Race and/or Ethnicity Among Adults in the United States, 2019–2023 eFigure 3. Trends in Age-Standardized Anxiety/Depression Prevalence by Disability Status and Sex Among Adults in the United States, 2019–2023 eFigure 4. Trends in Age-Standardized Anxiety/Depression Prevalence by Disability Status and Nativity Among Adults in the United States, 2019–2023 eFigure 5. Trends in Age-Standardized Anxiety/Depression Prevalence by Disability Status, Race and/or Ethnicity, Sex, and Nativity Among Adults in the United States, 2019–2023 eFigure 6. Trends in Age-Standardized Anxiety/Depression Prevalence by Vision Disability Status Among Adults in the United States, 2019–2023 eFigure 7. Trends in Age-Standardized Anxiety/Depression Prevalence by Vision Disability Status and Race and/or Ethnicity Among Adults in the United States, 2019–2023 eFigure 8. Trends in Age-Standardized Anxiety/Depression Prevalence by Vision Disability Status and Sex Among Adults in the United States, 2019–2023 eFigure 9. Trends in Age-Standardized Anxiety/Depression Prevalence by Vision Disability Status and Nativity Among Adults in the United States, 2019–2023 eFigure 10. Trends in Age-Standardized Anxiety/Depression Prevalence by Hearing Disability Status Among Adults in the United States, 2019–2023 eFigure 11. Trends in Age-Standardized Anxiety/Depression Prevalence by Hearing Disability Status and Race and/or Ethnicity Among Adults in the United States, 2019–2023 eFigure 12. Trends in Age-Standardized Anxiety/Depression Prevalence by Hearing Disability Status and Sex Among Adults in the United States, 2019–2023 eFigure 13. Trends in Age-Standardized Anxiety/Depression Prevalence by Hearing Disability Status and Nativity Among Adults in the United States, 2019–2023 eFigure 14. [file jamanetwopen-e2557332-s001.pdf]

Supplemental Online Content

Adzrago D, Fujimoto K, Wilkerson JM, Dyer TV, Williams F. Anxiety or depression trends by disability status and demographic intersections in US adults, 2019-2023. JAMA Netw Open. 2026;9(2):e2557332. doi:10.1001/jamanetworkopen.2025.57332

eFigure 1. Trends in Age-Standardized Anxiety/Depression Prevalence by Disability Status Among Adults in the United States, 2019–2023
eFigure 2. Trends in Age-Standardized Anxiety/Depression Prevalence by Disability Status and Race and/or Ethnicity Among Adults in the United States, 2019–2023
eFigure 3. Trends in Age-Standardized Anxiety/Depression Prevalence by Disability Status and Sex Among Adults in the United States, 2019–2023
eFigure 4. Trends in Age-Standardized Anxiety/Depression Prevalence by Disability Status and Nativity Among Adults in the United States, 2019–2023
eFigure 5. Trends in Age-Standardized Anxiety/Depression Prevalence by Disability Status, Race and/or Ethnicity, Sex, and Nativity Among Adults in the United States, 2019–2023
eFigure 6. Trends in Age-Standardized Anxiety/Depression Prevalence by Vision Disability Status Among Adults in the United States, 2019–2023
eFigure 7. Trends in Age-Standardized Anxiety/Depression Prevalence by Vision Disability Status and Race and/or Ethnicity Among Adults in the United States, 2019–2023
eFigure 8. Trends in Age-Standardized Anxiety/Depression Prevalence by Vision Disability Status and Sex Among Adults in the United States, 2019–2023
eFigure 9. Trends in Age-Standardized Anxiety/Depression Prevalence by Vision Disability Status and Nativity Among Adults in the United States, 2019–2023
eFigure 10. Trends in Age-Standardized Anxiety/Depression Prevalence by Hearing Disability Status Among Adults in the United States, 2019–2023
eFigure 11. Trends in Age-Standardized Anxiety/Depression Prevalence by Hearing Disability Status and Race and/or Ethnicity Among Adults in the United States, 2019–2023
eFigure 12. Trends in Age-Standardized Anxiety/Depression Prevalence by Hearing Disability Status and Sex Among Adults in the United States, 2019–2023
eFigure 13. Trends in Age-Standardized Anxiety/Depression Prevalence by Hearing Disability Status and Nativity Among Adults in the United States, 2019–2023
eFigure 14. Trends in Age-Standardized Anxiety/Depression Prevalence by Mobility Disability Status Among Adults in the United States, 2019–2023
eFigure 15. Trends in Age-Standardized Anxiety/Depression Prevalence by Mobility Disability Status and Race and/or Ethnicity Among Adults in the United States, 2019–2023
eFigure 16. Trends in Age-Standardized Anxiety/Depression Prevalence by Mobility Disability Status and Sex Among Adults in the United States, 2019–2023
eFigure 17. Trends in Age-Standardized Anxiety/Depression Prevalence by Mobility Disability Status and Nativity Among Adults in the United States, 2019–2023
eFigure 18. Trends in Age-Standardized Anxiety/Depression Prevalence by Communication Disability Status Among Adults in the United States, 2019–2023
eFigure 19. Trends in Age-Standardized Anxiety/Depression Prevalence by Communication Disability Status and Race and/or Ethnicity Among Adults in the United States, 2019–2023
eFigure 20. Trends in Age-Standardized Anxiety/Depression Prevalence by Communication Disability Status and Sex Among Adults in the United States, 2019–2023

© 2026 Adzrago D et al. *JAMA Network Open*.

eFigure 21. Trends in Age-Standardized Anxiety/Depression Prevalence by Communication Disability Status and Nativity Among Adults in the United States, 2019–2023
eFigure 22. Trends in Age-Standardized Anxiety/Depression Prevalence by Cognition Disability Status Among Adults in the United States, 2019–2023
eFigure 23. Trends in Age-Standardized Anxiety/Depression Prevalence by Cognition Disability Status and Race and/or Ethnicity Among Adults in the United States, 2019–2023
eFigure 24. Trends in Age-Standardized Anxiety/Depression Prevalence by Cognition Disability Status and Sex Among Adults in the United States, 2019–2023
eFigure 25. Trends in Age-Standardized Anxiety/Depression Prevalence by Cognition Disability Status and Nativity Among Adults in the United States, 2019–2023
eFigure 26. Trends in Age-Standardized Anxiety/Depression Prevalence by Self-care Disability Status Among Adults in the United States, 2019–2023
eFigure 27. Trends in Age-Standardized Anxiety/Depression Prevalence by Self-care Disability Status and Race and/or Ethnicity Among Adults in the United States, 2019–2023
eFigure 28. Trends in Age-Standardized Anxiety/Depression Prevalence by Self-care Disability Status and Sex Among Adults in the United States, 2019–2023
eFigure 29. Trends in Age-Standardized Anxiety/Depression Prevalence by Self-care Disability Status and Nativity Among Adults in the United States, 2019–2023
eTable 1. Anxiety/Depression Prevalence by Disability Status and Survey Year Among Adults in the United States
eTable 2. Trends in Anxiety/Depression by Disability Status Among Adults in the United States, 2019–2023
eTable 3. Trends in Anxiety/Depression by Vision Disability Status Among Adults in the United States, 2019–2023
eTable 4. Trends in Anxiety/Depression by Race and/or Ethnicity and Vision Disability Status Among Adults in the United States, 2019–2023
eTable 5. Trends in Anxiety/Depression by Sex and Vision Disability Status Among Adults in the United States, 2019–2023
eTable 6. Trends in Anxiety/Depression by Nativity and Vision Disability Status Among Adults in the United States, 2019–2023
eTable 7. Trends in Anxiety/Depression by Hearing Disability Status Among Adults in the United States, 2019–2023
eTable 8. Trends in Anxiety/Depression by Race and/or Ethnicity and Hearing Disability Status Among Adults in the United States, 2019–2023
eTable 9. Trends in Anxiety/Depression by Sex and Hearing Disability Status Among Adults in the United States, 2019–2023
eTable 10. Trends in Anxiety/Depression by Nativity and Hearing Disability Status Among Adults in the United States, 2019–2023
eTable 11. Trends in Anxiety/Depression by Mobility Disability Status Among Adults in the United States, 2019–2023
eTable 12. Trends in Anxiety/Depression by Race and/or Ethnicity and Mobility Disability Status Among Adults in the United States, 2019–2023
eTable 13. Trends in Anxiety/Depression by Sex and Mobility Disability Status Among Adults in the United States, 2019–2023
eTable 14. Trends in Anxiety/Depression by Nativity and Mobility Disability Status Among Adults in the United States, 2019–2023
eTable 15. Trends in Anxiety/Depression by Communication Disability Status Among Adults in the United States, 2019–2023
eTable 16. Trends in Anxiety/Depression by Race and/or Ethnicity and Communication Disability Status Among Adults in the United States, 2019–2023

© 2026 Adzrago D et al. *JAMA Network Open*.

eTable 17. Trends in Anxiety/Depression by Sex and Communication Disability Status Among Adults in the United States, 2019–2023
eTable 18. Trends in Anxiety/Depression by Nativity and Communication Disability Status Among Adults in the United States, 2019–2023
eTable 19. Trends in Anxiety/Depression by Cognition Disability Status Among Adults in the United States, 2019–2023
eTable 20. Trends in Anxiety/Depression by Race and/or Ethnicity and Cognition Disability Status Among Adults in the United States, 2019–2023
eTable 21. Trends in Anxiety/Depression by Sex and Cognition Disability Status Among Adults in the United States, 2019–2023
eTable 22. Trends in Anxiety/Depression by Nativity and Cognition Disability Status Among Adults in the United States, 2019–2023
eTable 23. Trends in Anxiety/Depression by Self-care Disability Status Among Adults in the United States, 2019–2023
eTable 24. Trends in Anxiety/Depression by Race and/or Ethnicity and Self-care Disability Status Among Adults in the United States, 2019–2023
eTable 25. Trends in Anxiety/Depression by Sex and Self-care Disability Status Among Adults in the United States, 2019–2023
eTable 26. Trends in Anxiety/Depression by Nativity and Self-care Disability Status Among Adults in the United States, 2019–2023

This supplemental material has been provided by the authors to give readers additional information about their work.

© 2026 Adzrago D et al. *JAMA Network Open*.

Overall Disabilities

eFigure 1. Trends in Age-Standardized Anxiety/Depression Prevalence by Disability Status Among Adults in the United States, 2019–2023

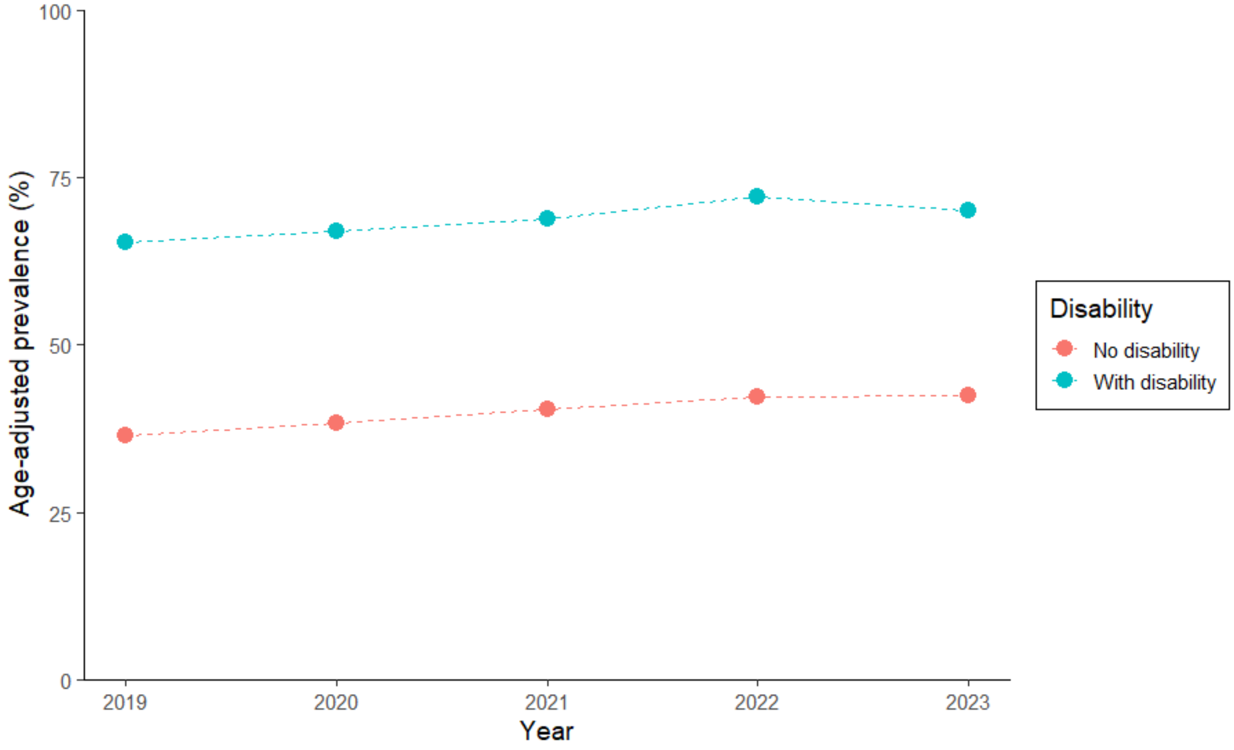

© 2026 Adzrago D et al. *JAMA Network Open*.

eFigure 2. Trends in Age-Standardized Anxiety/Depression Prevalence by Disability Status and Race and/or Ethnicity Among Adults in the United States, 2019–2023

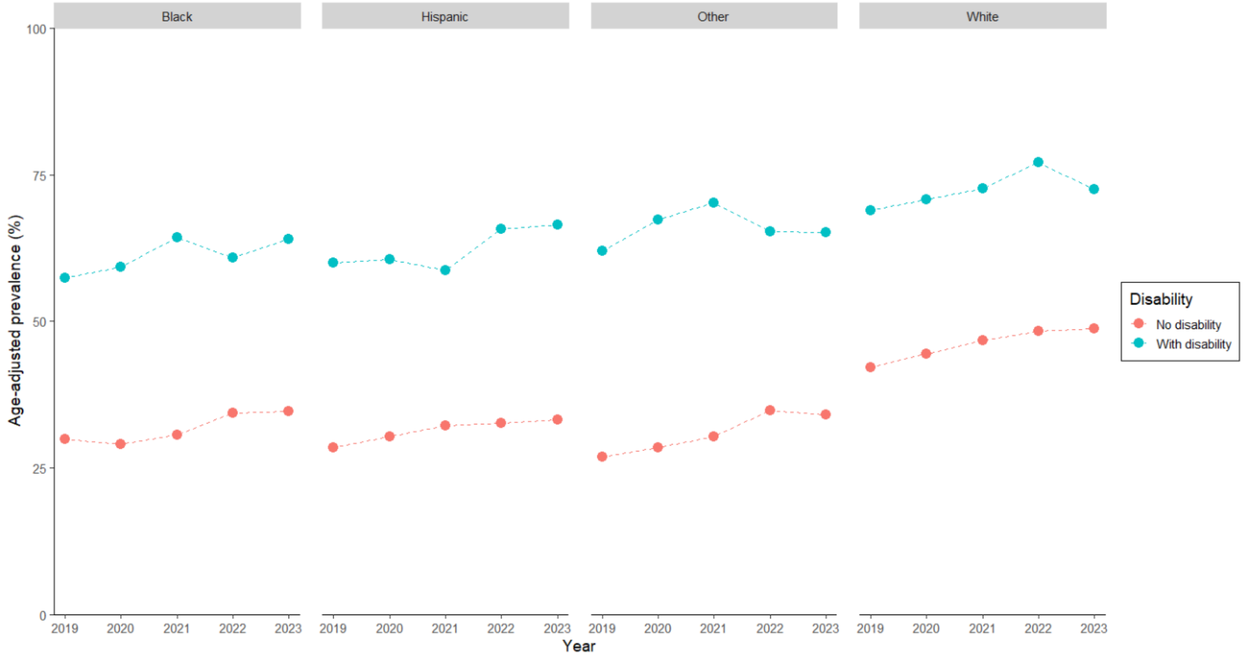

© 2026 Adzrago D et al. *JAMA Network Open*.

eFigure 3. Trends in Age-Standardized Anxiety/Depression Prevalence by Disability Status and Sex Among Adults in the United States, 2019–2023

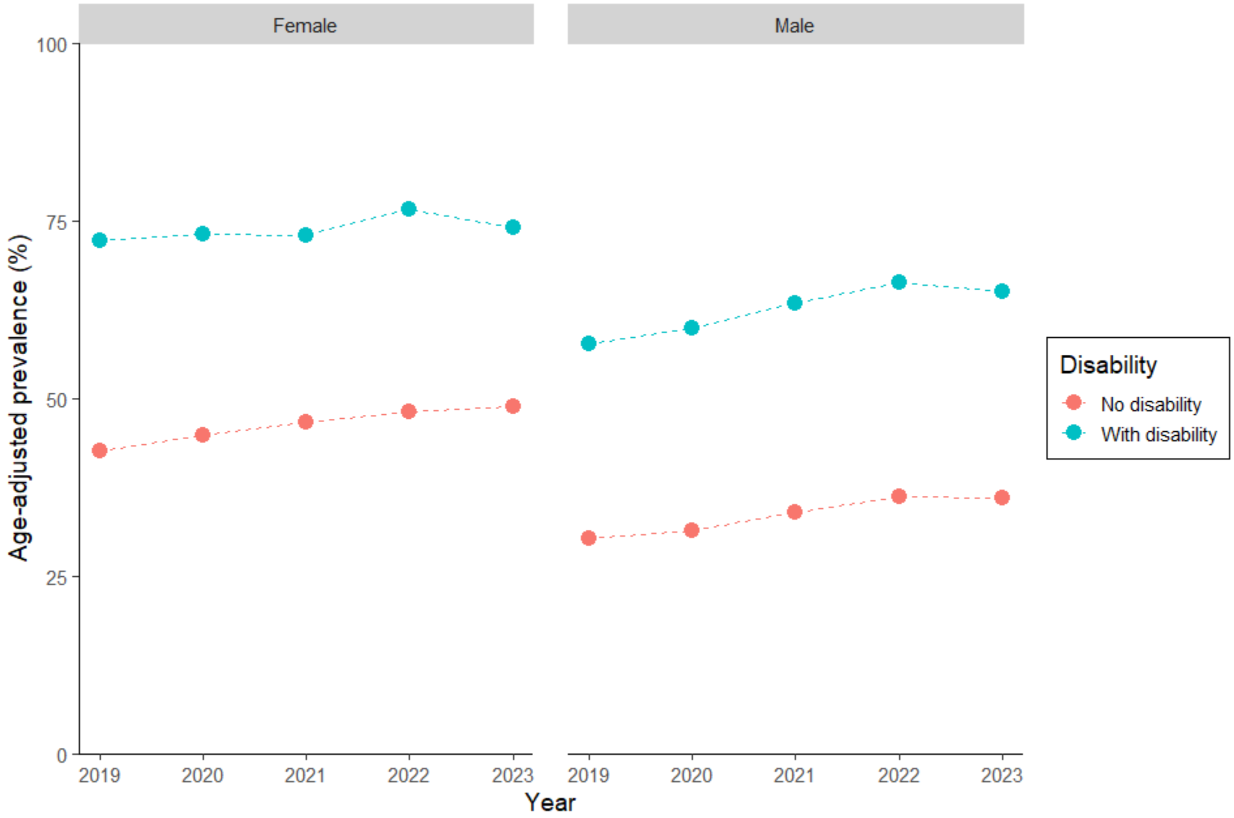

© 2026 Adzrago D et al. *JAMA Network Open*.

eFigure 4. Trends in Age-Standardized Anxiety/Depression Prevalence by Disability Status and Nativity Among Adults in the United States, 2019–2023

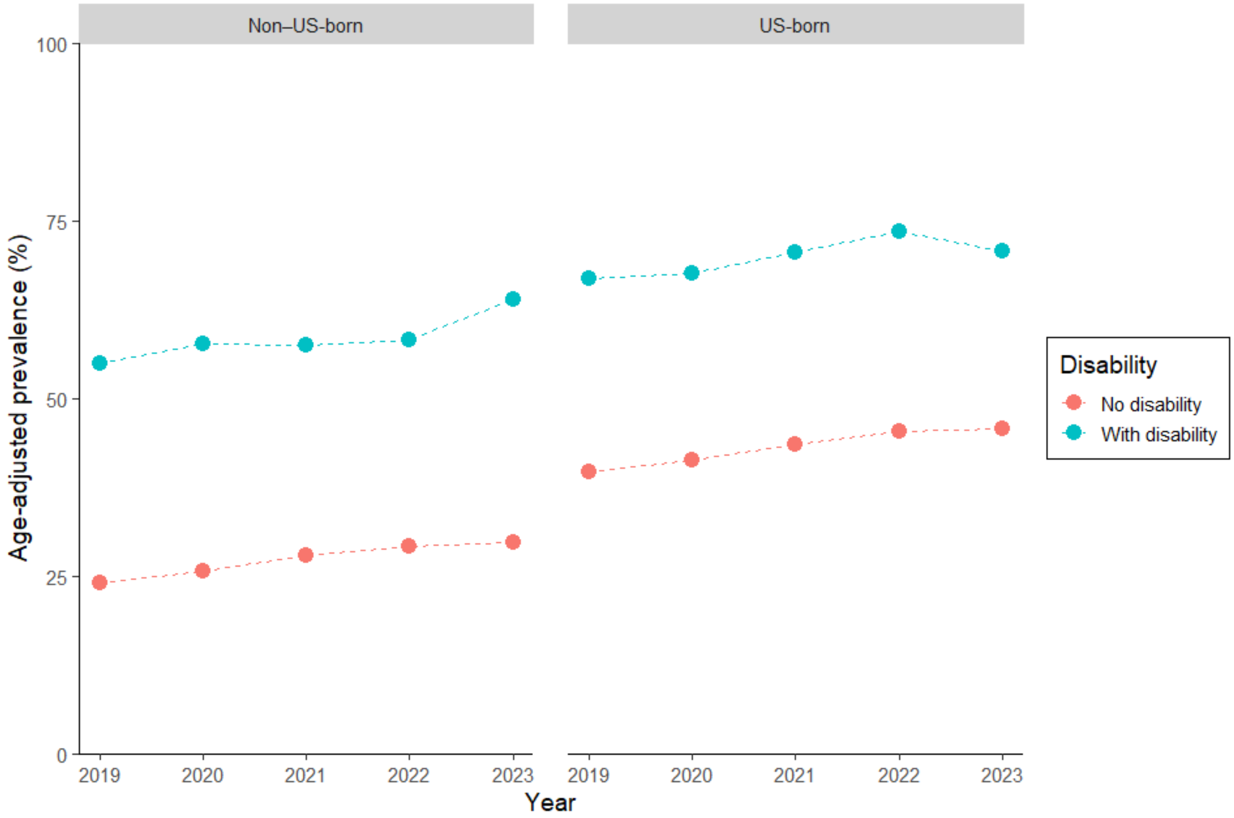

© 2026 Adzrago D et al. *JAMA Network Open*.

eFigure 5. Trends in Age-Standardized Anxiety/Depression Prevalence by Disability Status, Race and/or Ethnicity, Sex, and Nativity Among Adults in the United States, 2019–2023

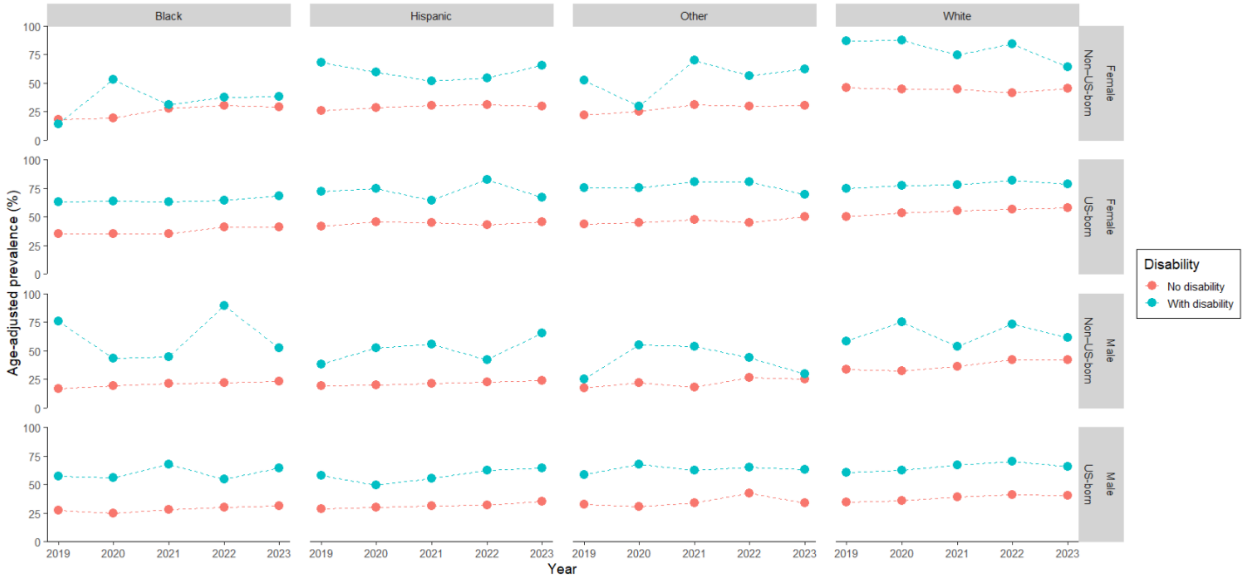

© 2026 Adzrago D et al. *JAMA Network Open*.

Six Disability Domains

Vision Disability Status

eFigure 6. Trends in Age-Standardized Anxiety/Depression Prevalence by Vision Disability Status Among Adults in the United States, 2019–2023

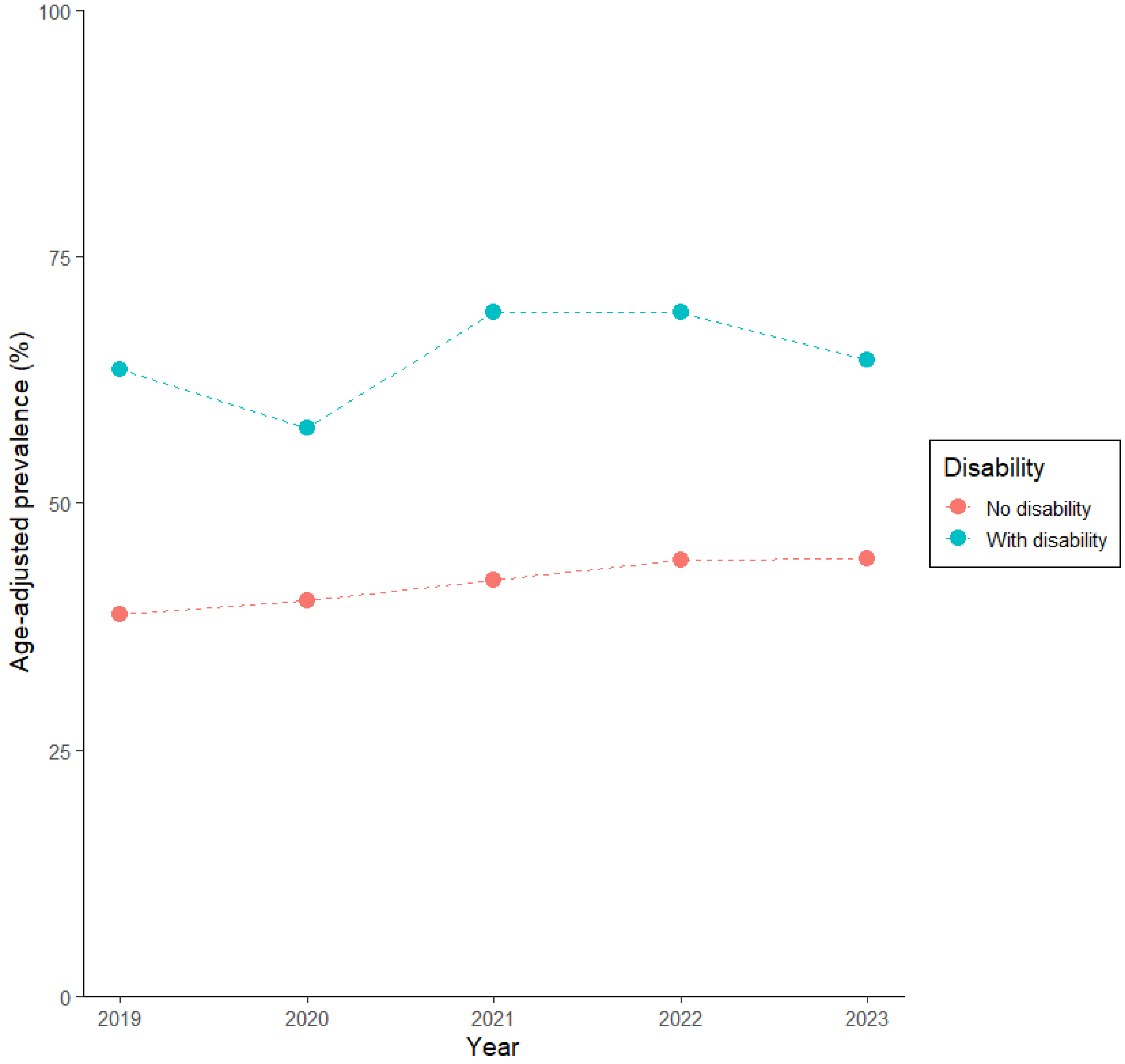

© 2026 Adzrago D et al. *JAMA Network Open*.

eFigure 7. Trends in Age-Standardized Anxiety/Depression Prevalence by Vision Disability Status and Race and/or Ethnicity Among Adults in the United States, 2019–2023

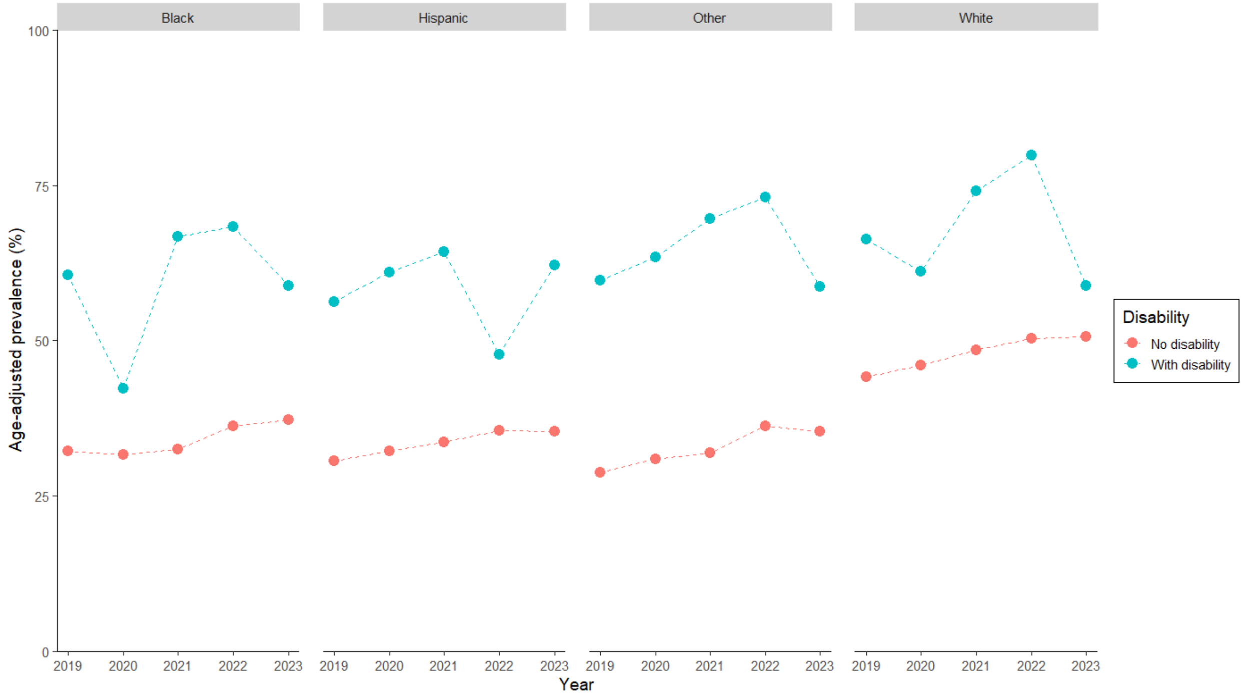

© 2026 Adzrago D et al. *JAMA Network Open*.

eFigure 8. Trends in Age-Standardized Anxiety/Depression Prevalence by Vision Disability Status and Sex Among Adults in the United States, 2019–2023

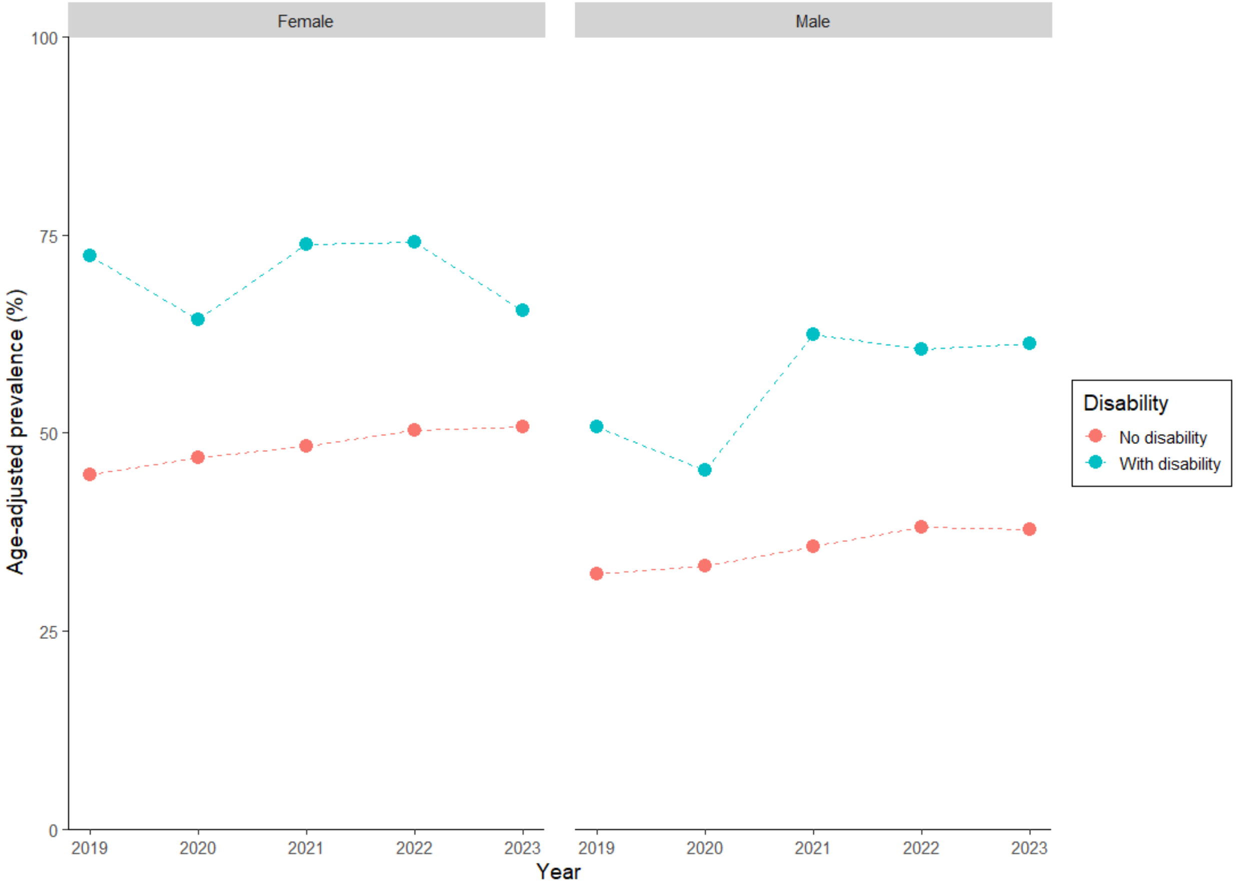

© 2026 Adzrago D et al. *JAMA Network Open*.

eFigure 9. Trends in Age-Standardized Anxiety/Depression Prevalence by Vision Disability Status and Nativity Among Adults in the United States, 2019–2023

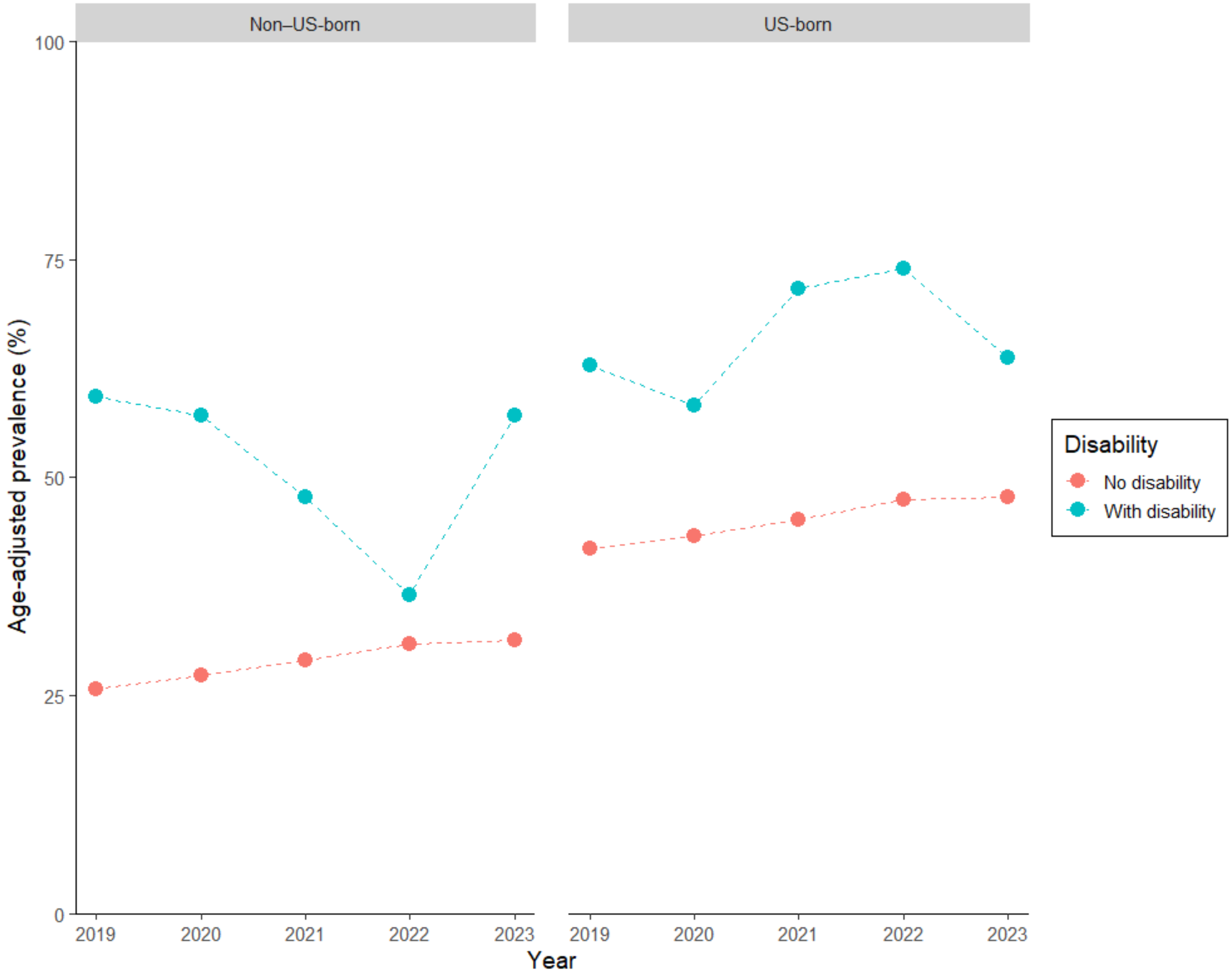

© 2026 Adzrago D et al. *JAMA Network Open*.

Hearing Disability Status

eFigure 10. Trends in Age-Standardized Anxiety/Depression Prevalence by Hearing Disability Status Among Adults in the United States, 2019–2023

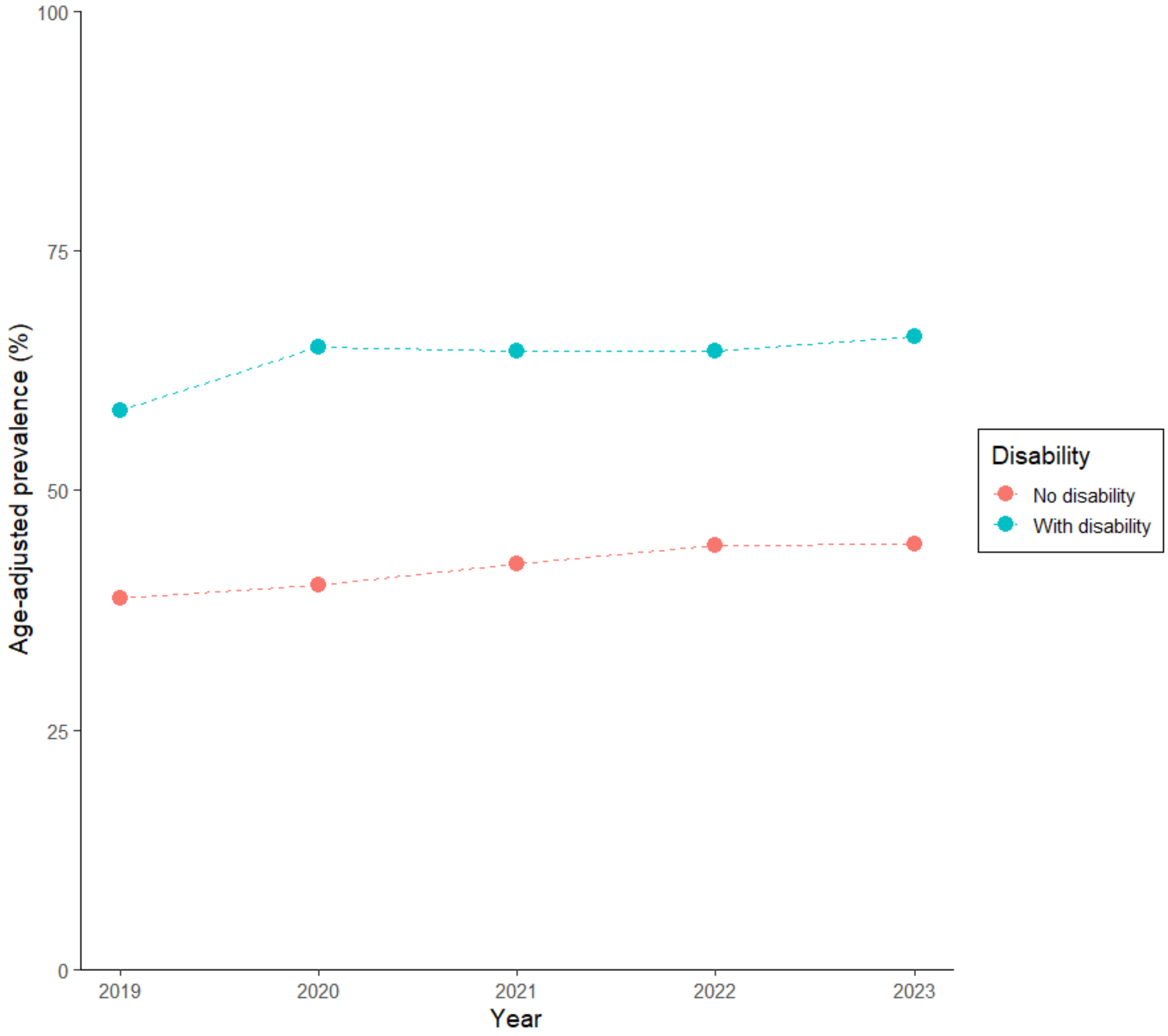

© 2026 Adzrago D et al. *JAMA Network Open*.

eFigure 11. Trends in Age-Standardized Anxiety/Depression Prevalence by Hearing Disability Status and Race and/or Ethnicity Among Adults in the United States, 2019–2023

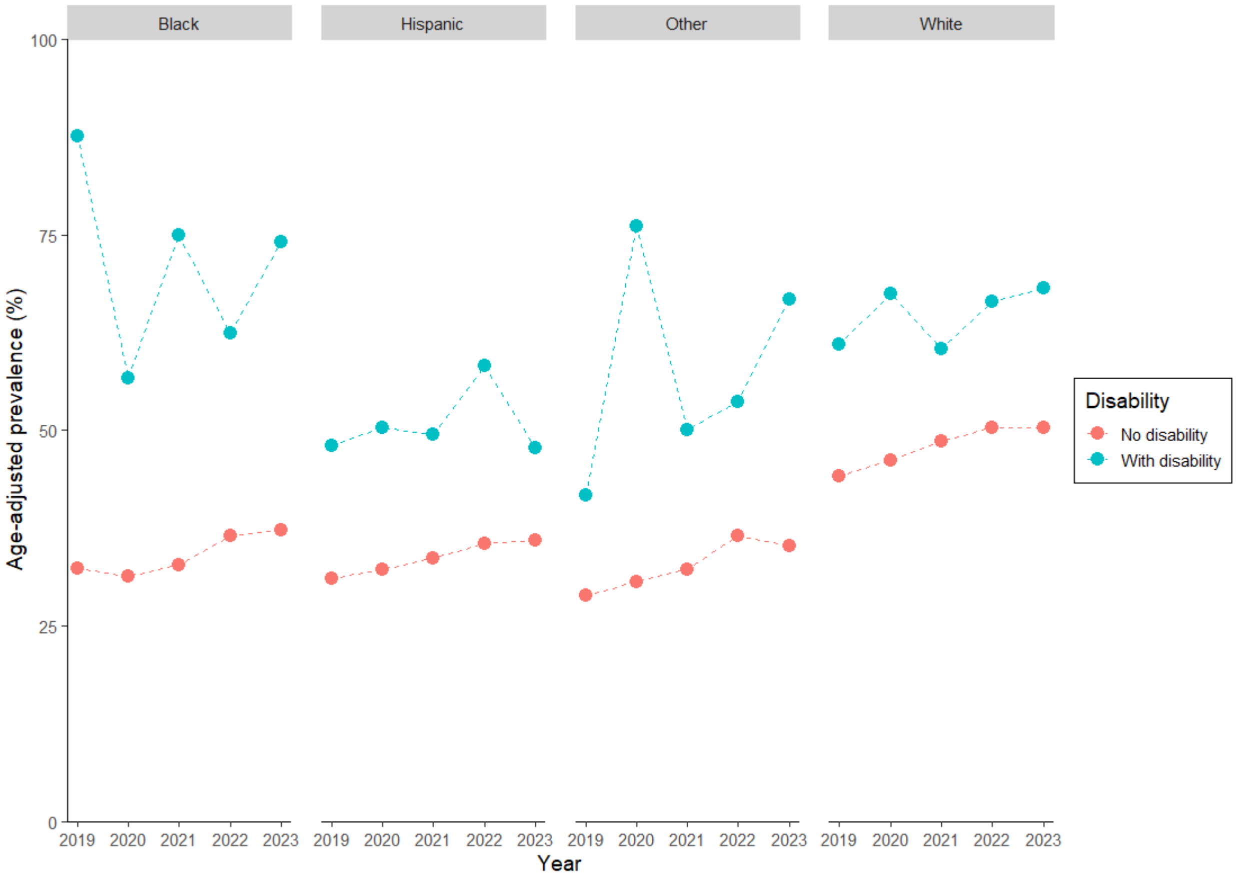

© 2026 Adzrago D et al. *JAMA Network Open*.

eFigure 12. Trends in Age-Standardized Anxiety/Depression Prevalence by Hearing Disability Status and Sex Among Adults in the United States, 2019–2023

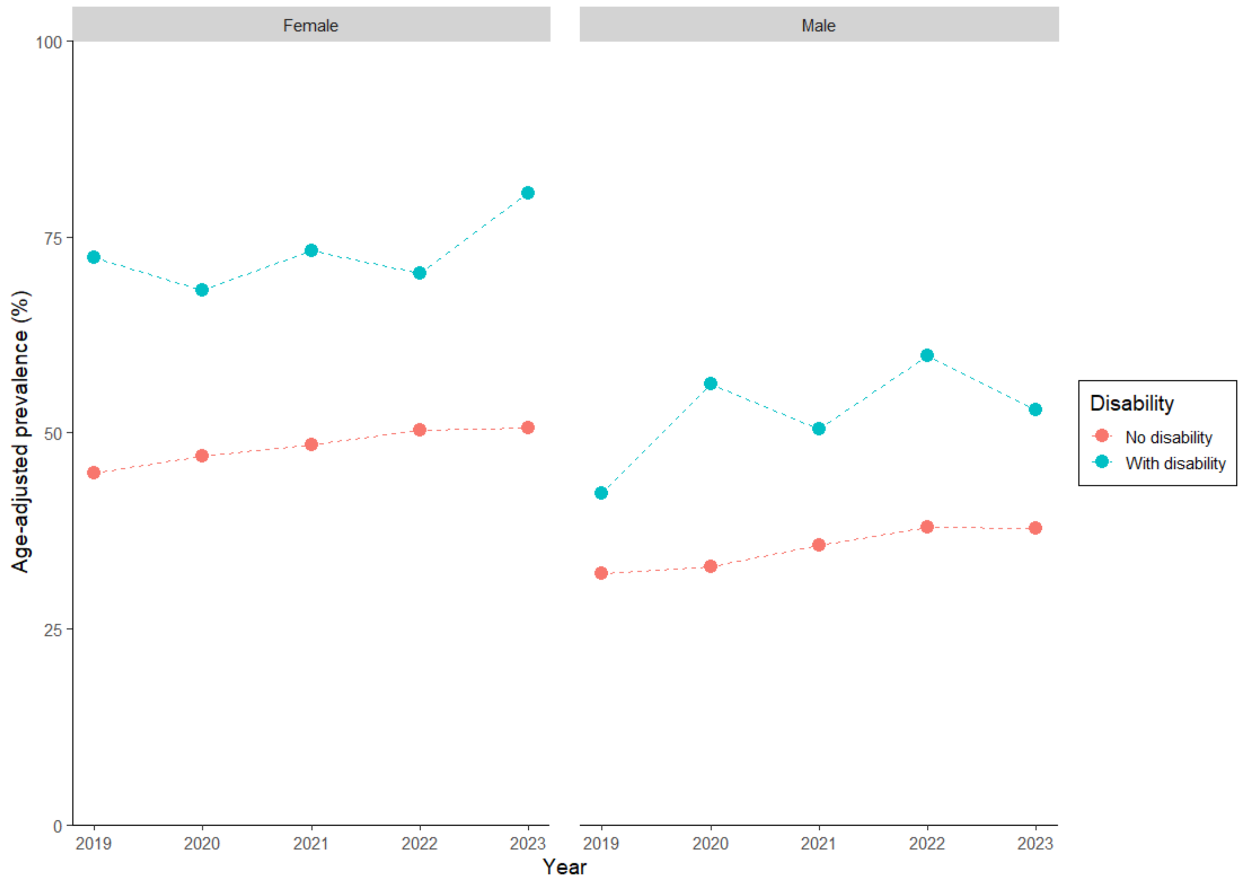

© 2026 Adzrago D et al. *JAMA Network Open*.

eFigure 13. Trends in Age-Standardized Anxiety/Depression Prevalence by Hearing Disability Status and Nativity Among Adults in the United States, 2019–2023

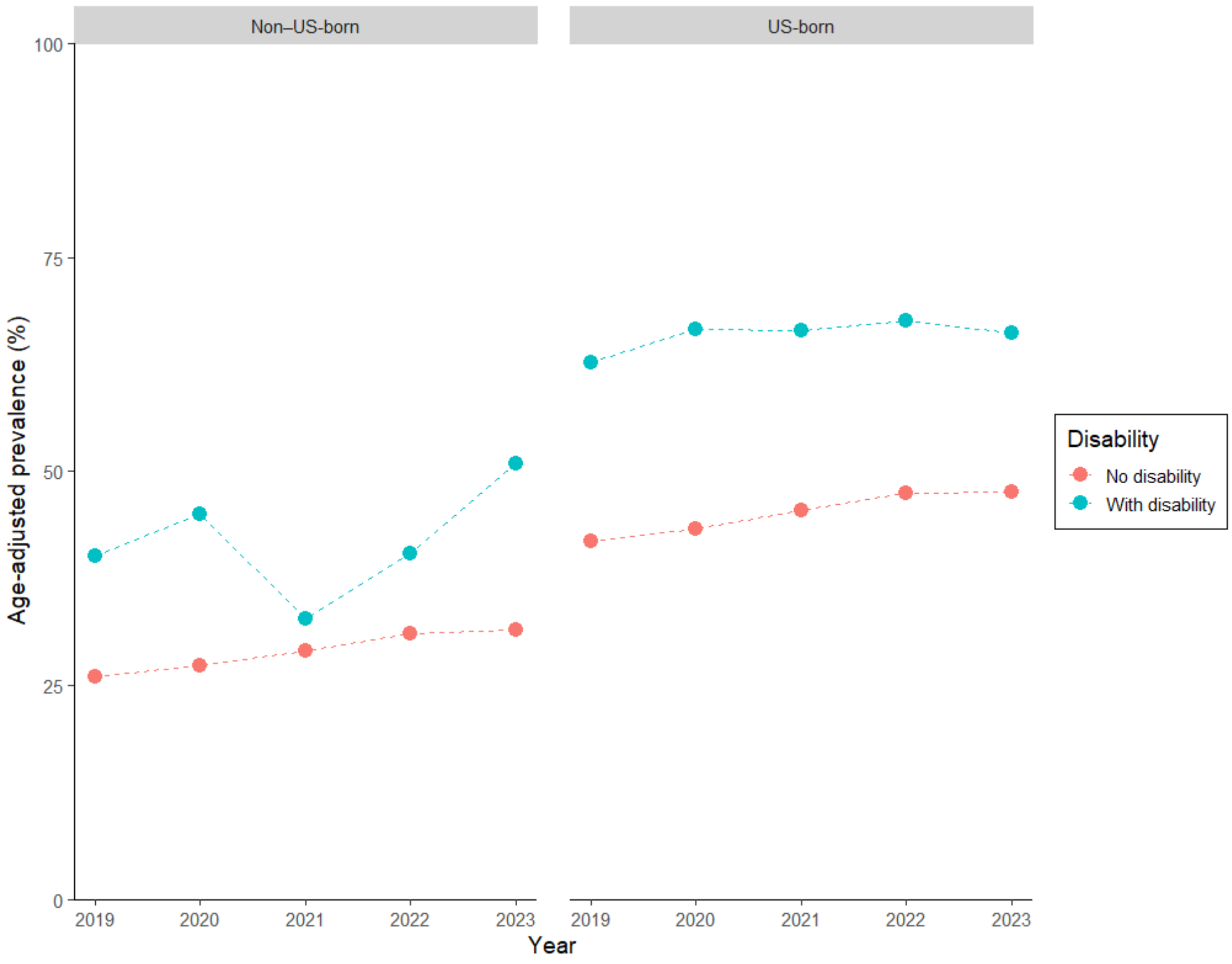

© 2026 Adzrago D et al. *JAMA Network Open*.

Mobility Disability Status

eFigure 14. Trends in Age-Standardized Anxiety/Depression Prevalence by Mobility Disability Status Among Adults in the United States, 2019–2023

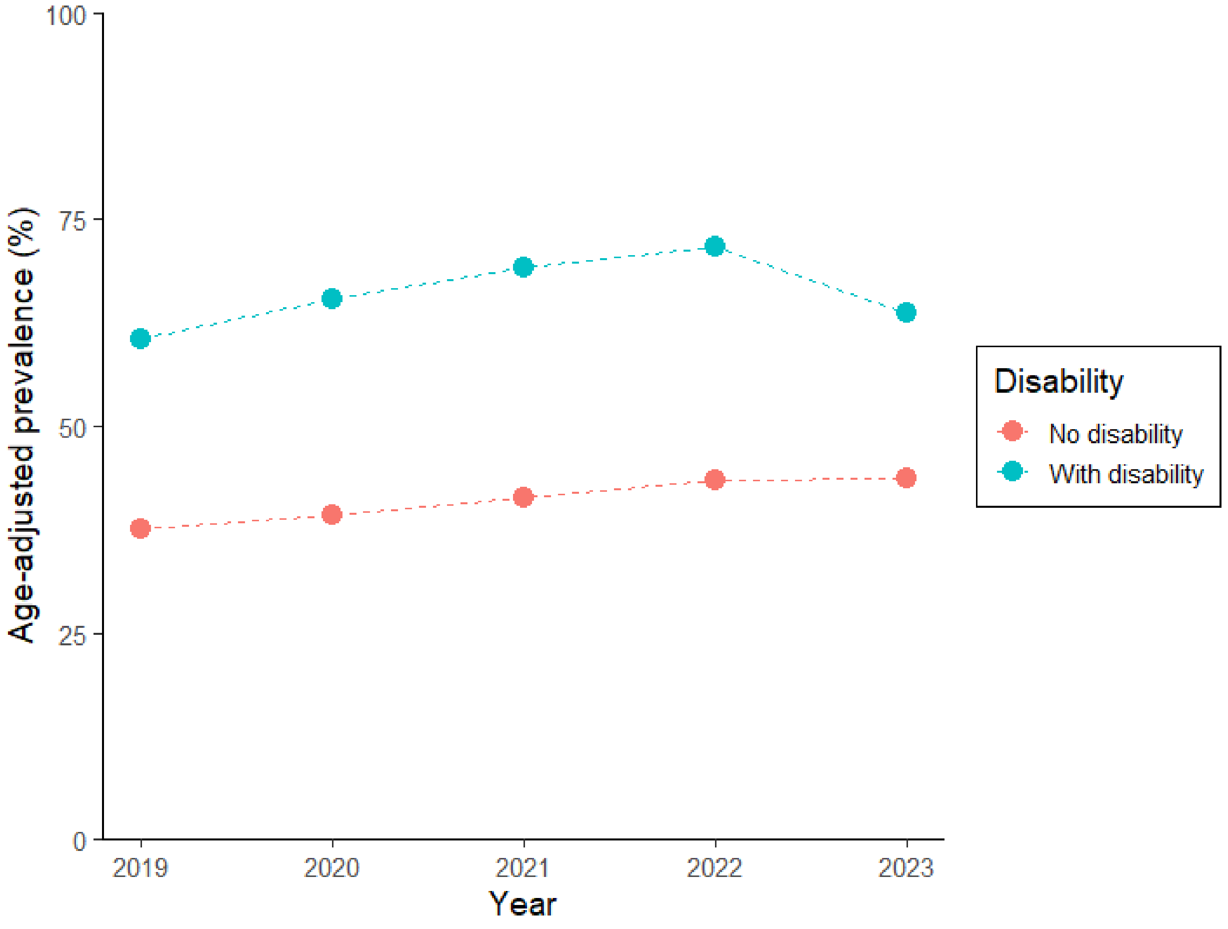

© 2026 Adzrago D et al. *JAMA Network Open*.

eFigure 15. Trends in Age-Standardized Anxiety/Depression Prevalence by Mobility Disability Status and Race and/or Ethnicity Among Adults in the United States, 2019–2023

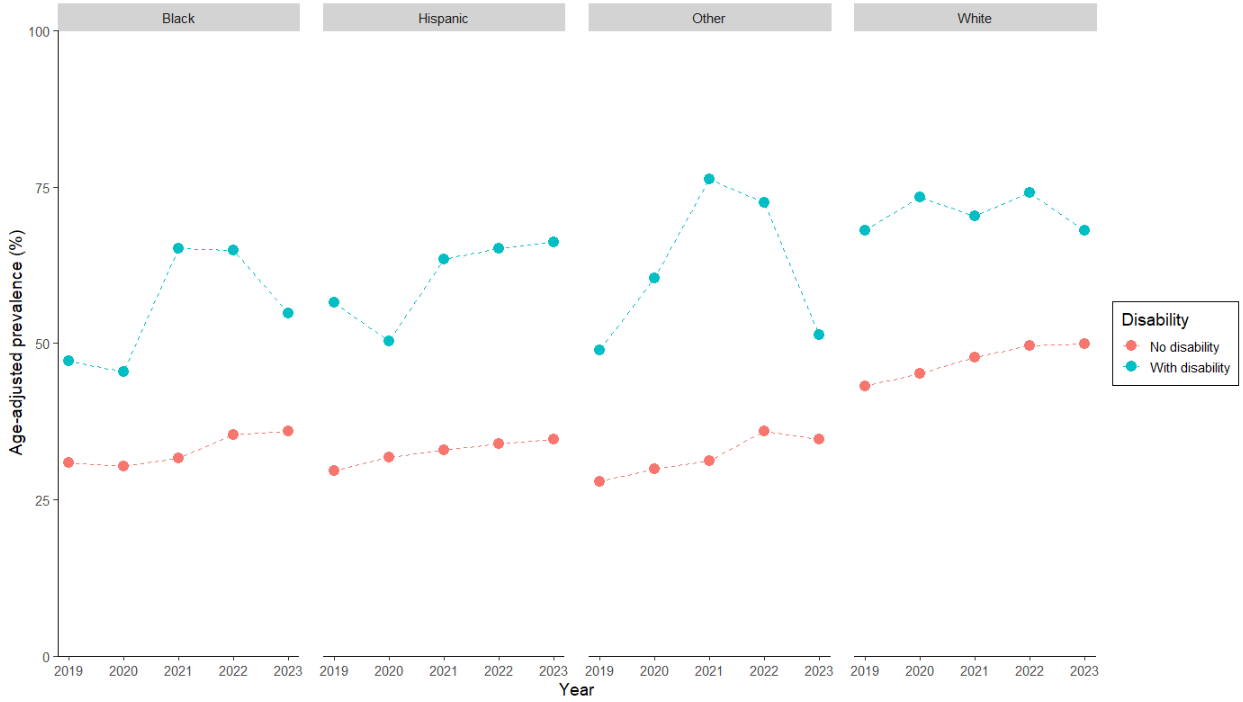

© 2026 Adzrago D et al. *JAMA Network Open*.

eFigure 16. Trends in Age-Standardized Anxiety/Depression Prevalence by Mobility Disability Status and Sex Among Adults in the United States, 2019–2023

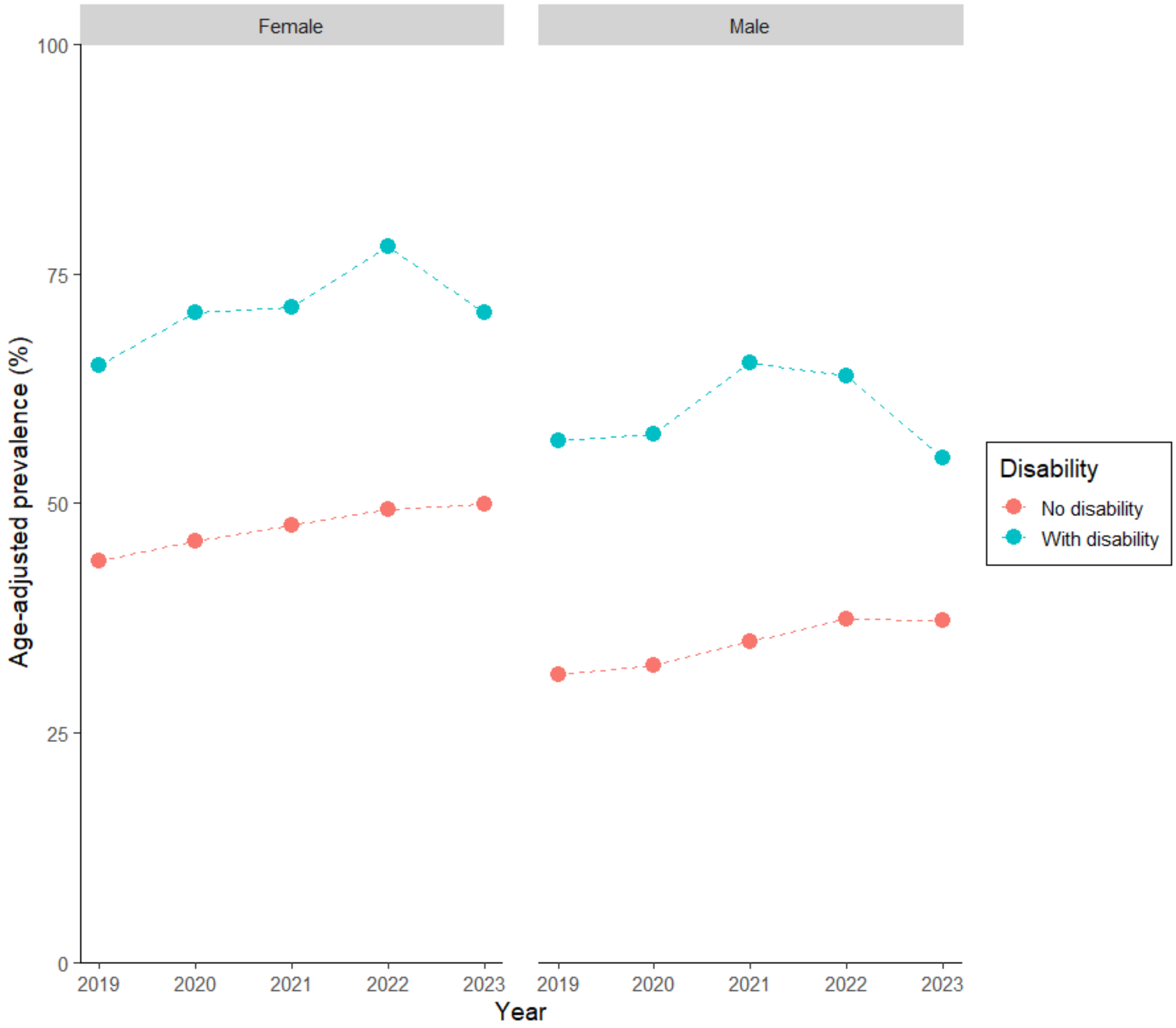

© 2026 Adzrago D et al. *JAMA Network Open*.

eFigure 17. Trends in Age-Standardized Anxiety/Depression Prevalence by Mobility Disability Status and Nativity Among Adults in the United States, 2019–2023

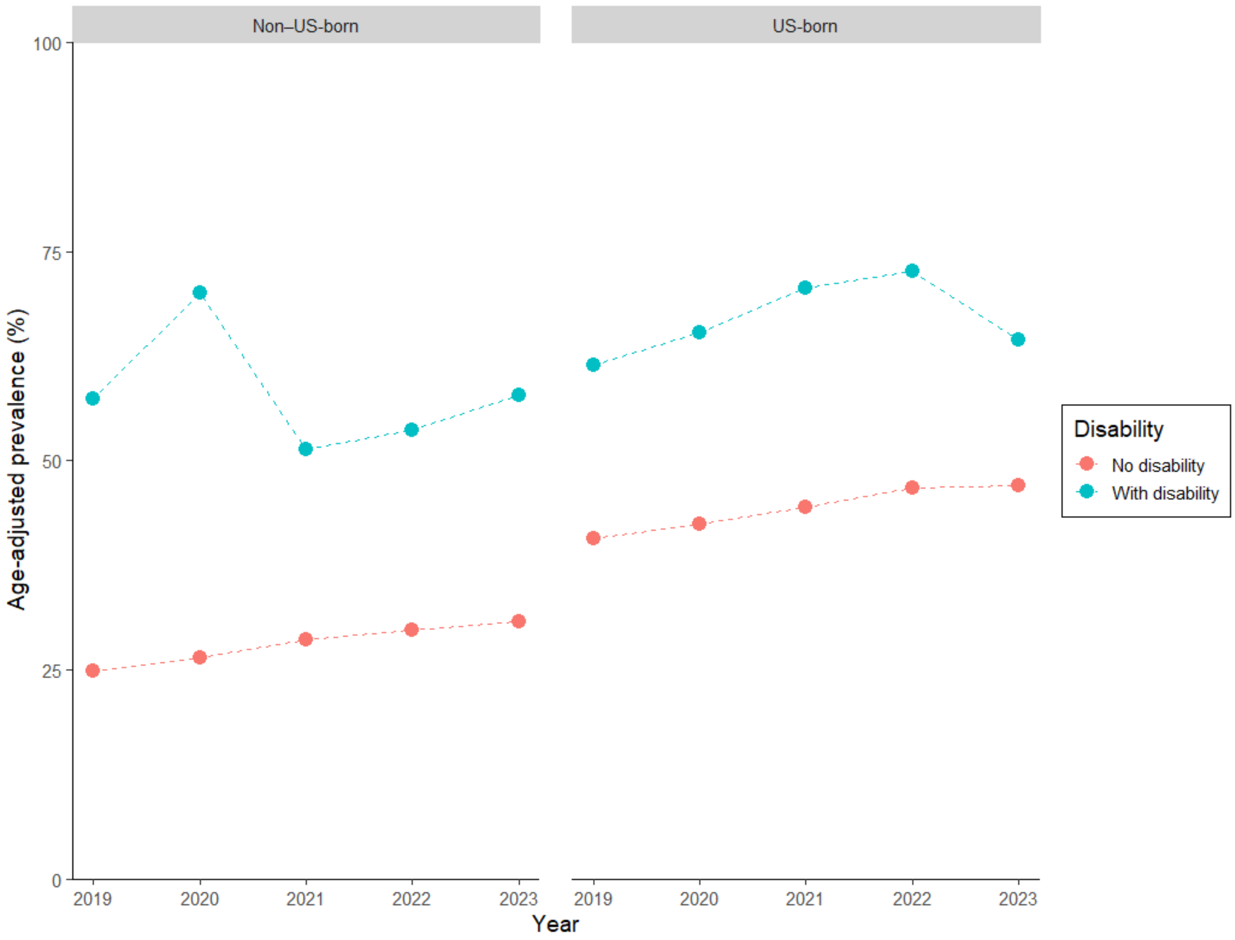

© 2026 Adzrago D et al. *JAMA Network Open*.

Communication Disability Status

eFigure 18. Trends in Age-Standardized Anxiety/Depression Prevalence by Communication Disability Status Among Adults in the United States, 2019–2023

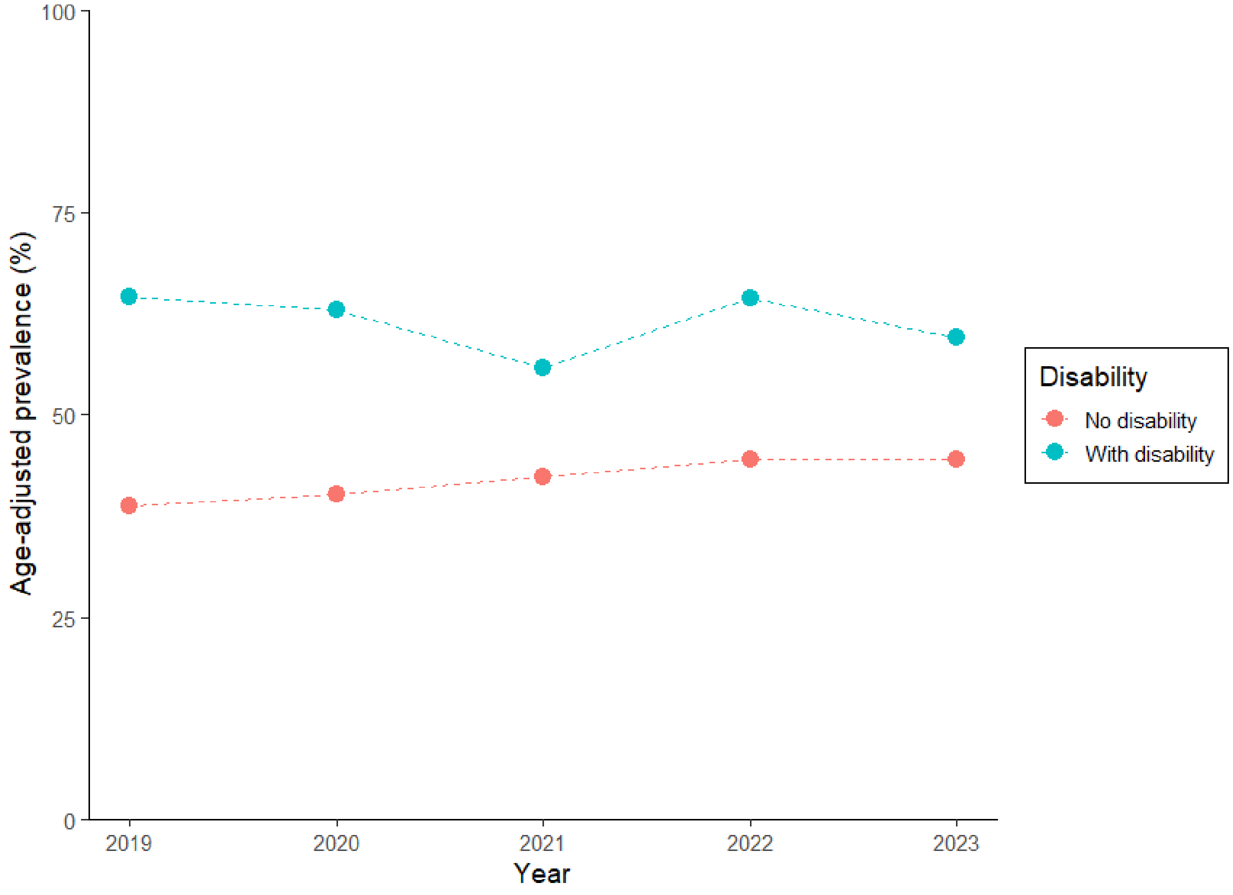

© 2026 Adzrago D et al. *JAMA Network Open*.

eFigure 19. Trends in Age-Standardized Anxiety/Depression Prevalence by Communication Disability Status and Race and/or Ethnicity Among Adults in the United States, 2019–2023

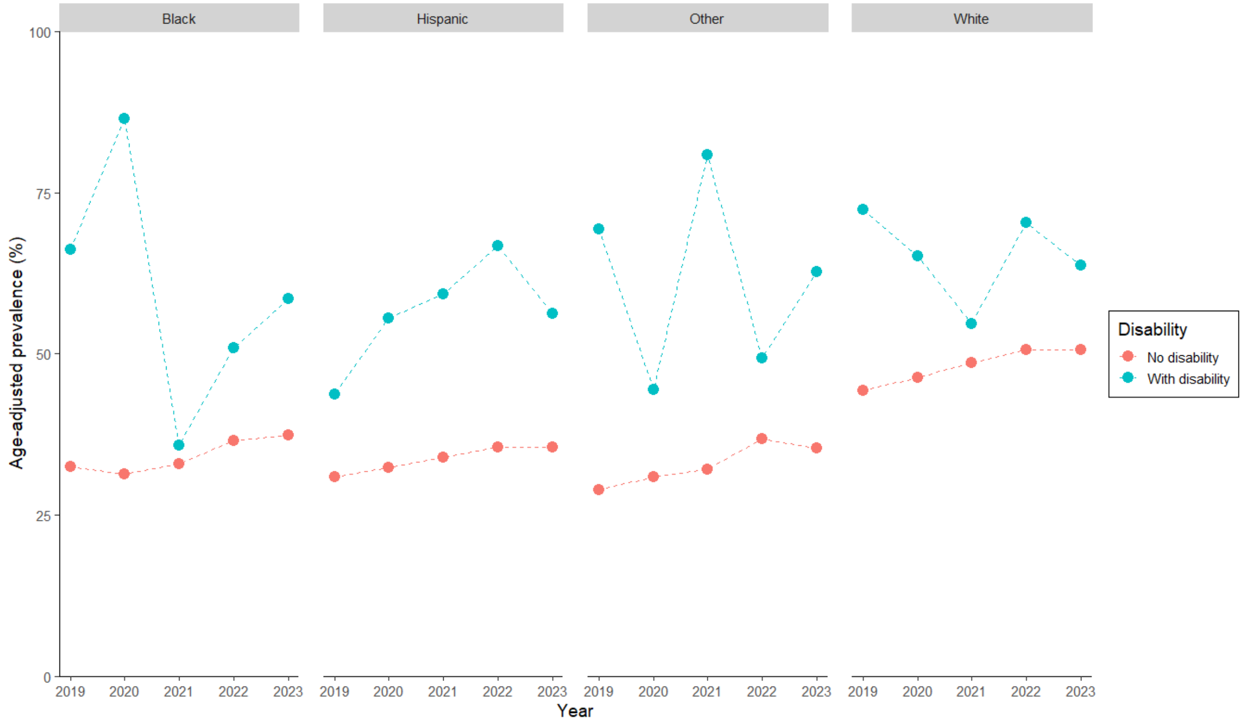

© 2026 Adzrago D et al. *JAMA Network Open*.

eFigure 20. Trends in Age-Standardized Anxiety/Depression Prevalence by Communication Disability Status and Sex Among Adults in the United States, 2019–2023

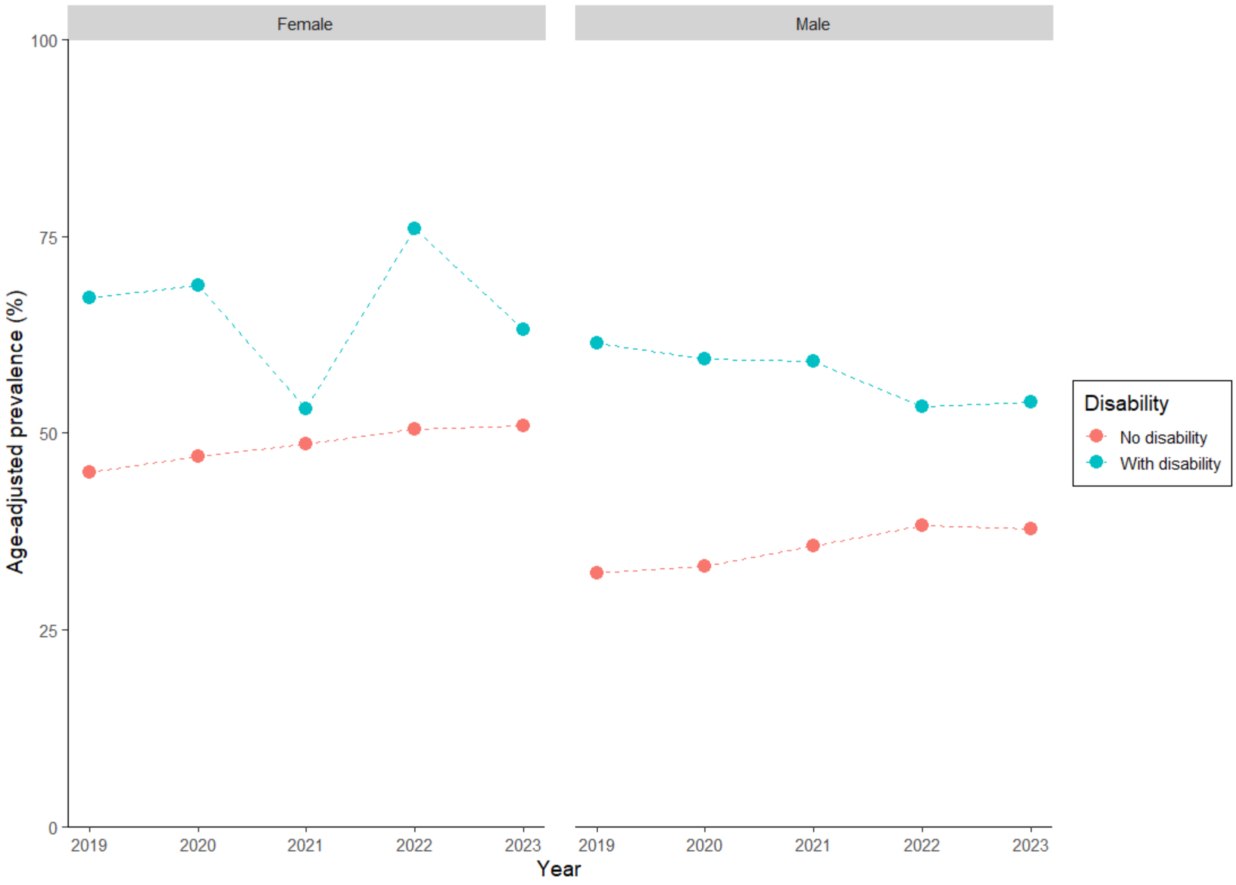

© 2026 Adzrago D et al. *JAMA Network Open*.

eFigure 21. Trends in Age-Standardized Anxiety/Depression Prevalence by Communication Disability Status and Nativity Among Adults in the United States, 2019–2023

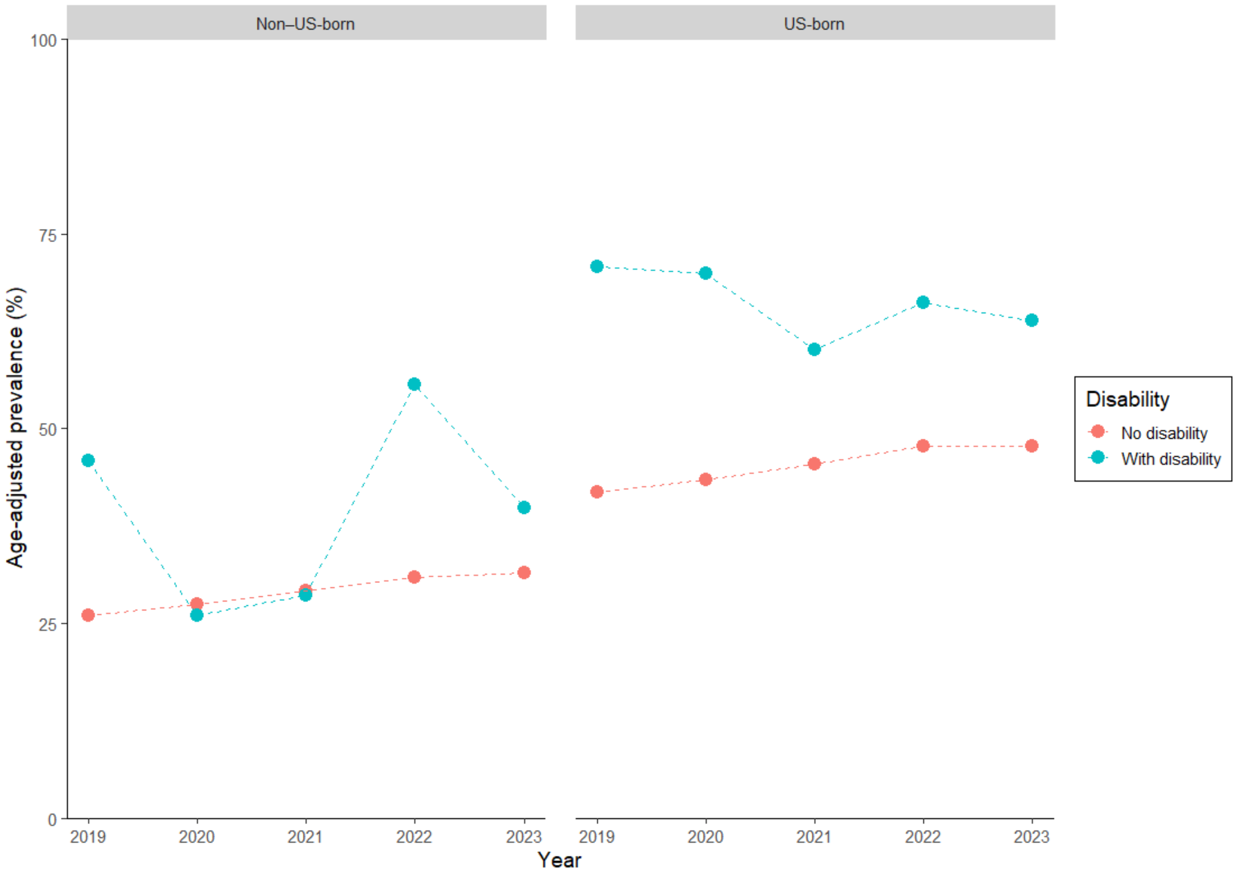

© 2026 Adzrago D et al. *JAMA Network Open*.

Cognition Disability Status

eFigure 22. Trends in Age-Standardized Anxiety/Depression Prevalence by Cognition Disability Status Among Adults in the United States, 2019–2023

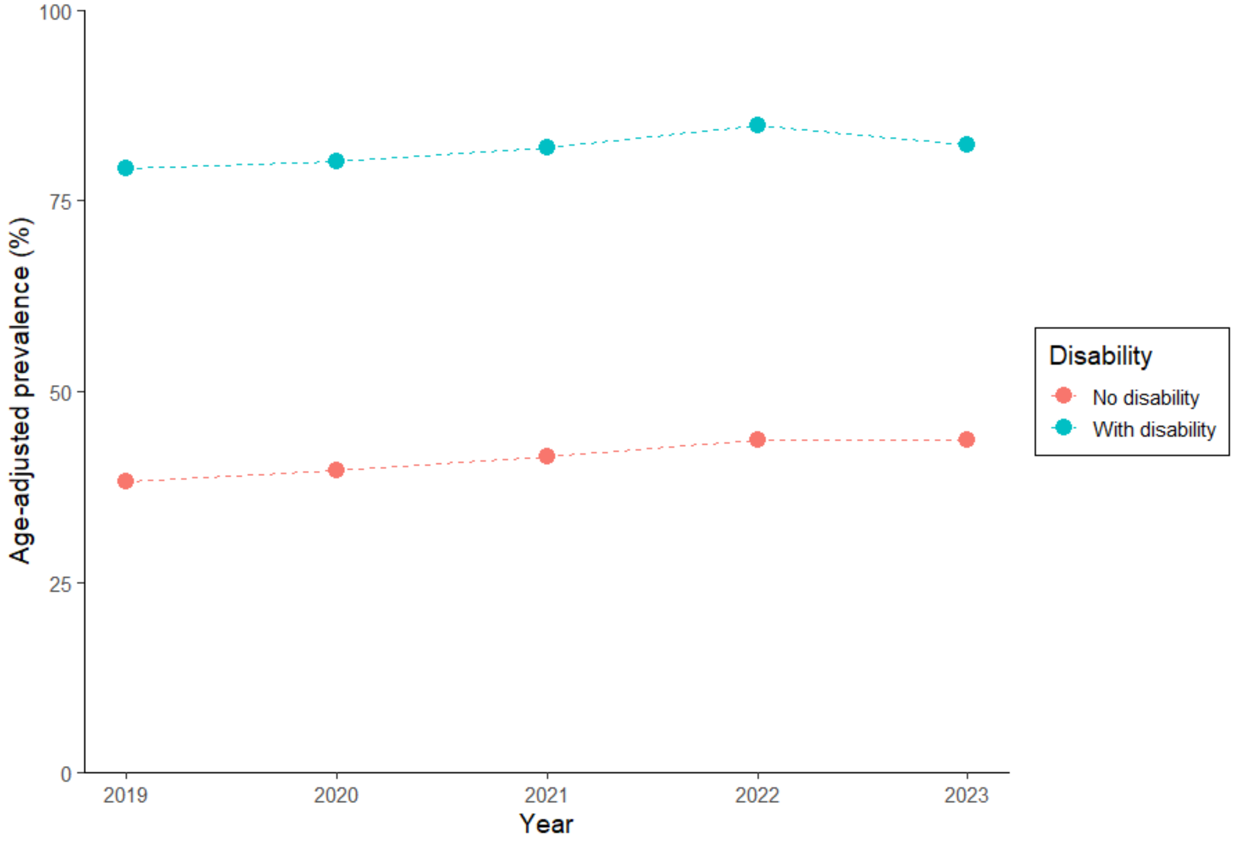

© 2026 Adzrago D et al. *JAMA Network Open*.

eFigure 23. Trends in Age-Standardized Anxiety/Depression Prevalence by Cognition Disability Status and Race and/or Ethnicity Among Adults in the United States, 2019–2023

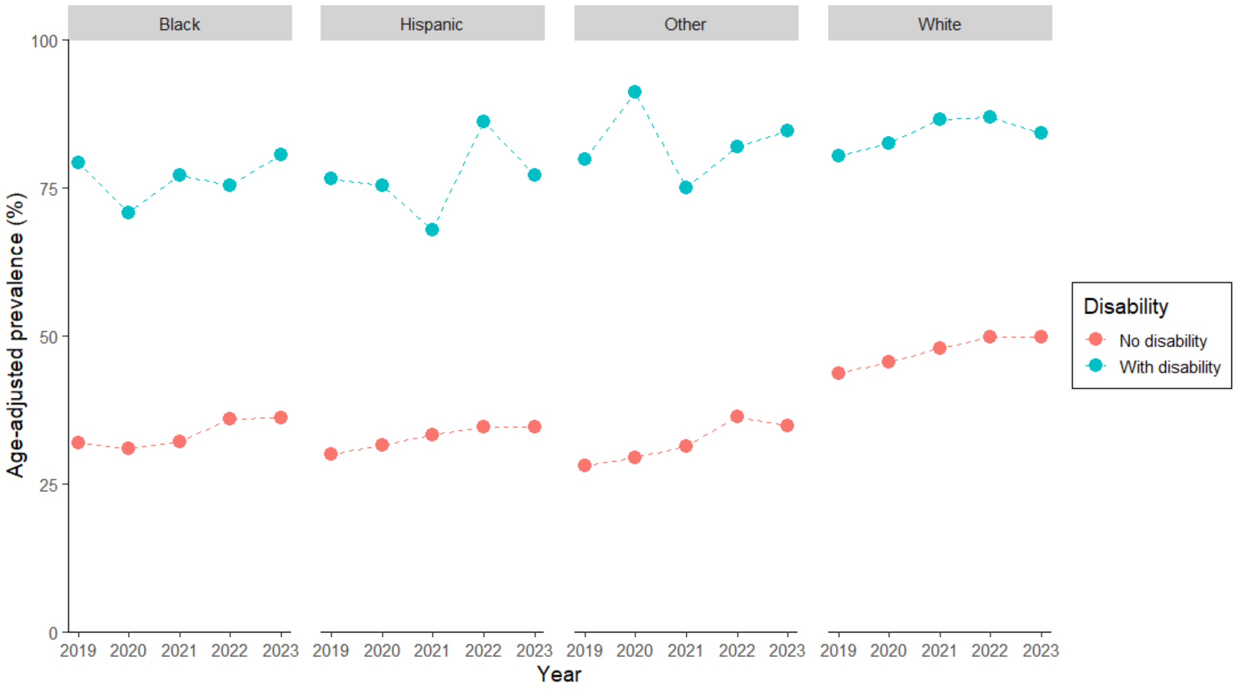

© 2026 Adzrago D et al. *JAMA Network Open*.

eFigure 24. Trends in Age-Standardized Anxiety/Depression Prevalence by Cognition Disability Status and Sex Among Adults in the United States, 2019–2023

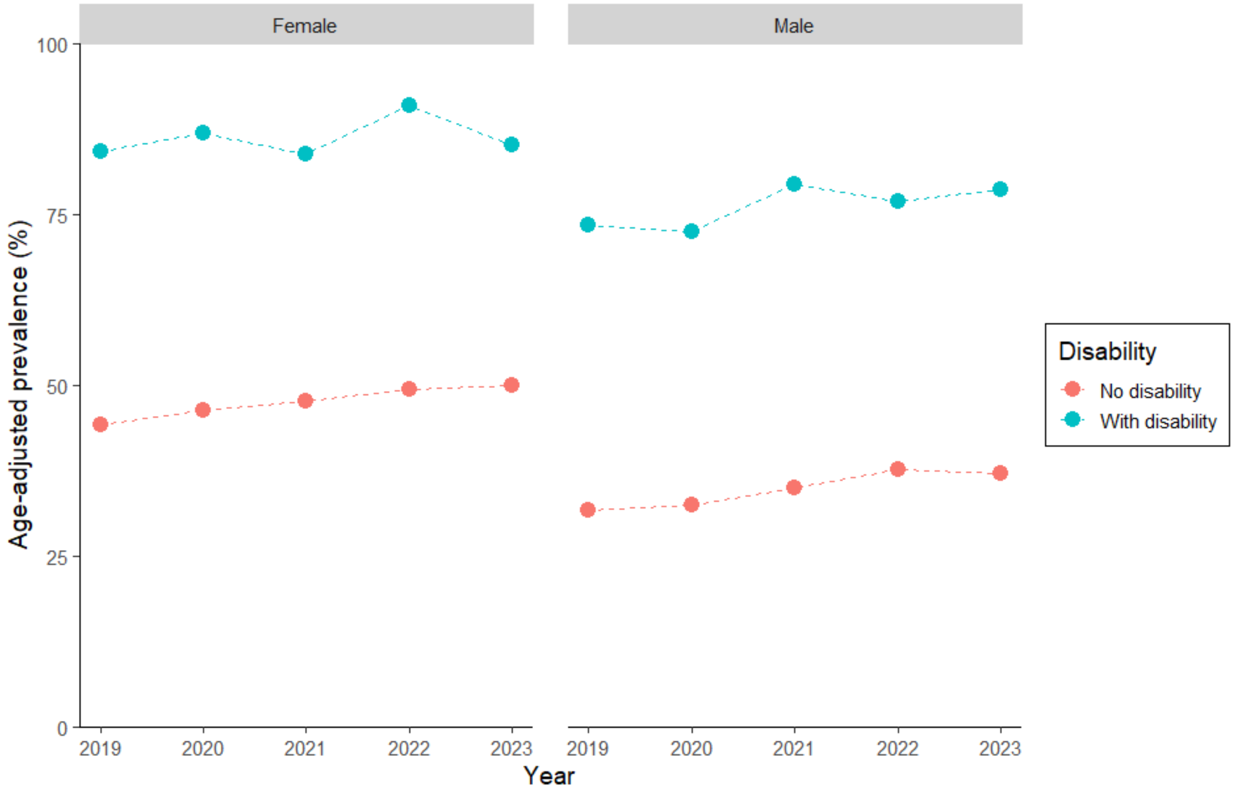

© 2026 Adzrago D et al. *JAMA Network Open*.

eFigure 25. Trends in Age-Standardized Anxiety/Depression Prevalence by Cognition Disability Status and Nativity Among Adults in the United States, 2019–2023

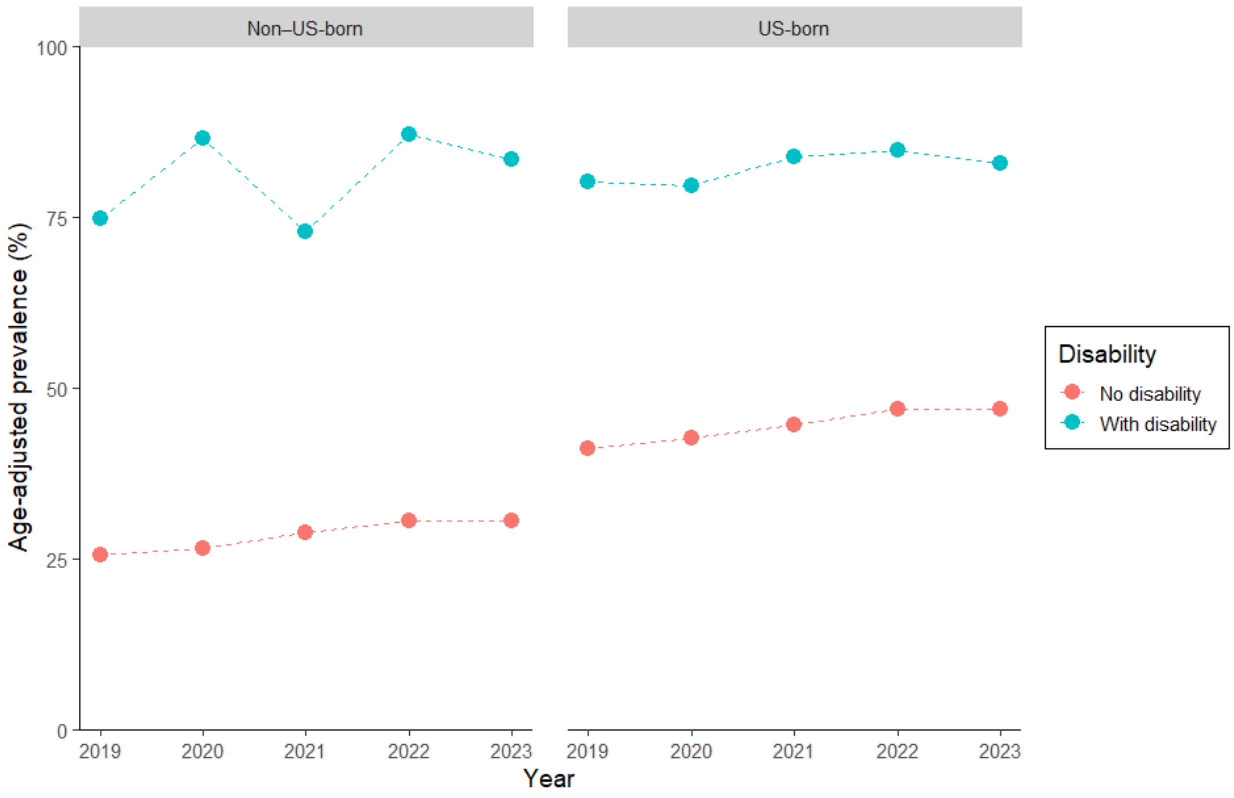

© 2026 Adzrago D et al. *JAMA Network Open*.

Self-care Disability Status

eFigure 26. Trends in Age-Standardized Anxiety/Depression Prevalence by Self-care Disability Status Among Adults in the United States, 2019–2023

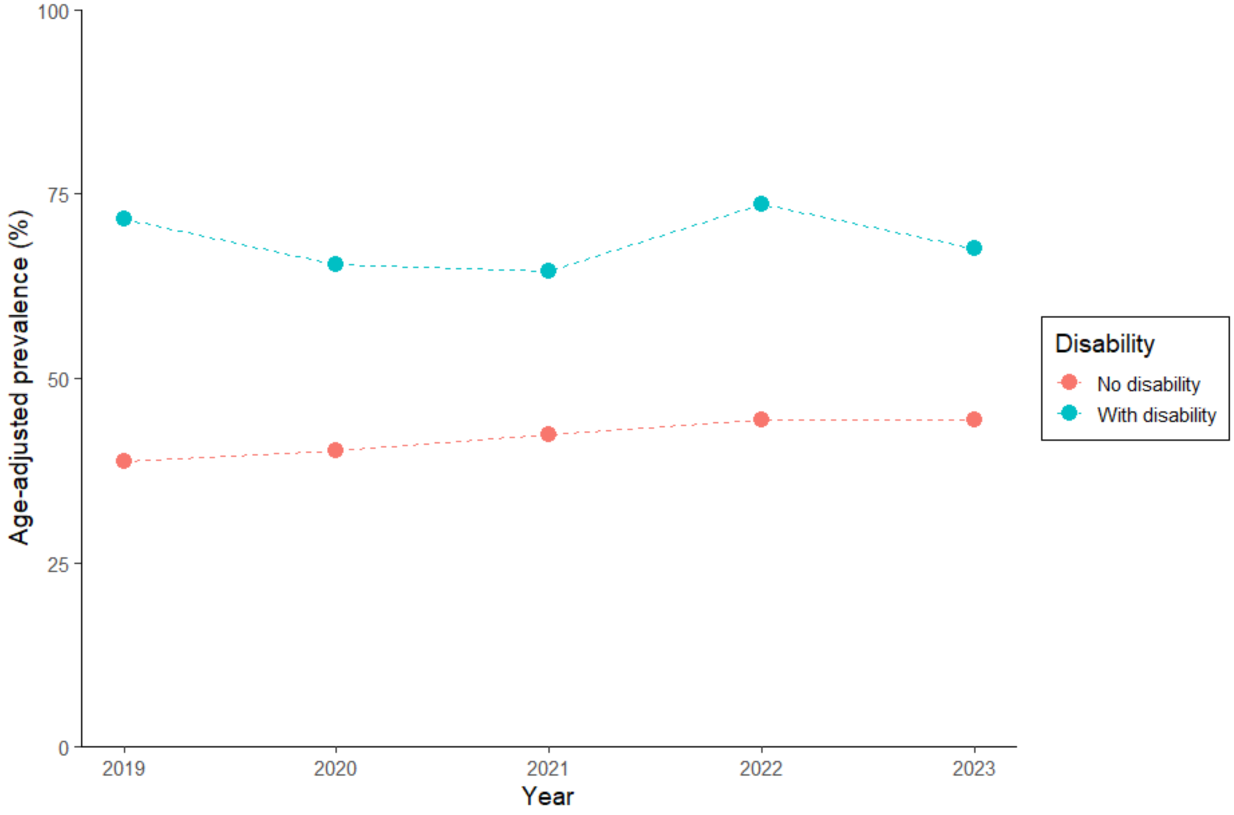

© 2026 Adzrago D et al. *JAMA Network Open*.

eFigure 27. Trends in Age-Standardized Anxiety/Depression Prevalence by Self-care Disability Status and Race and/or Ethnicity Among Adults in the United States, 2019–2023

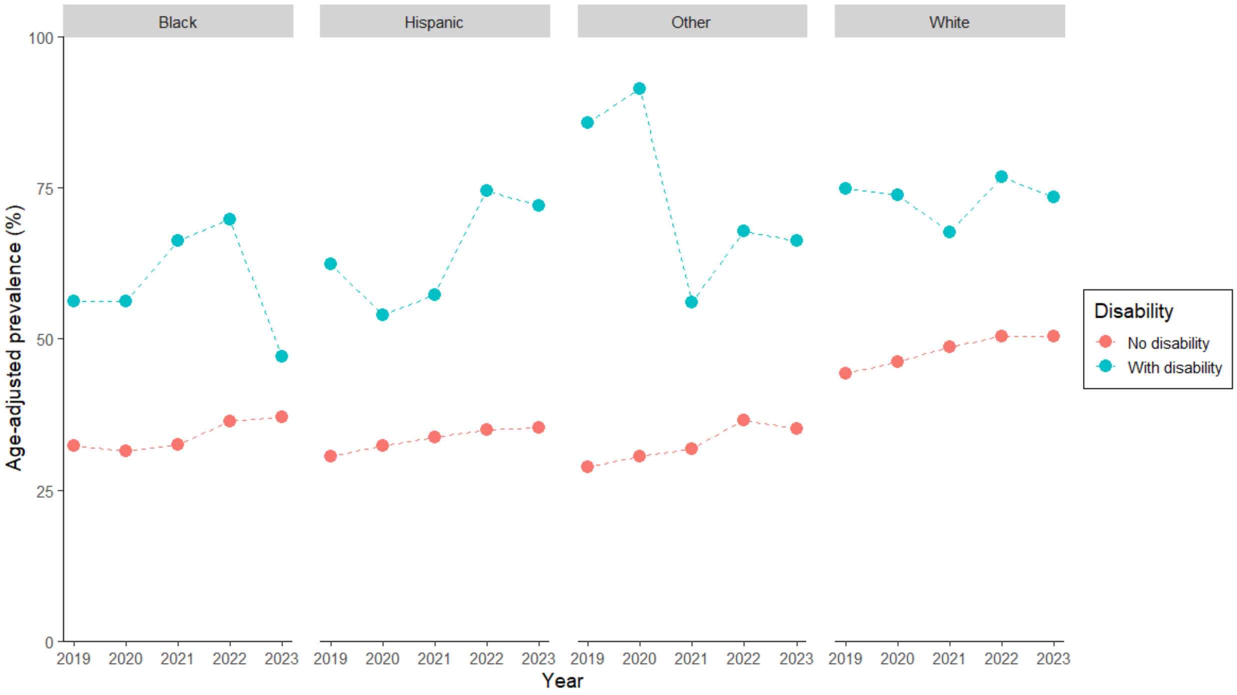

© 2026 Adzrago D et al. *JAMA Network Open*.

eFigure 28. Trends in Age-Standardized Anxiety/Depression Prevalence by Self-care Disability Status and Sex Among Adults in the United States, 2019–2023

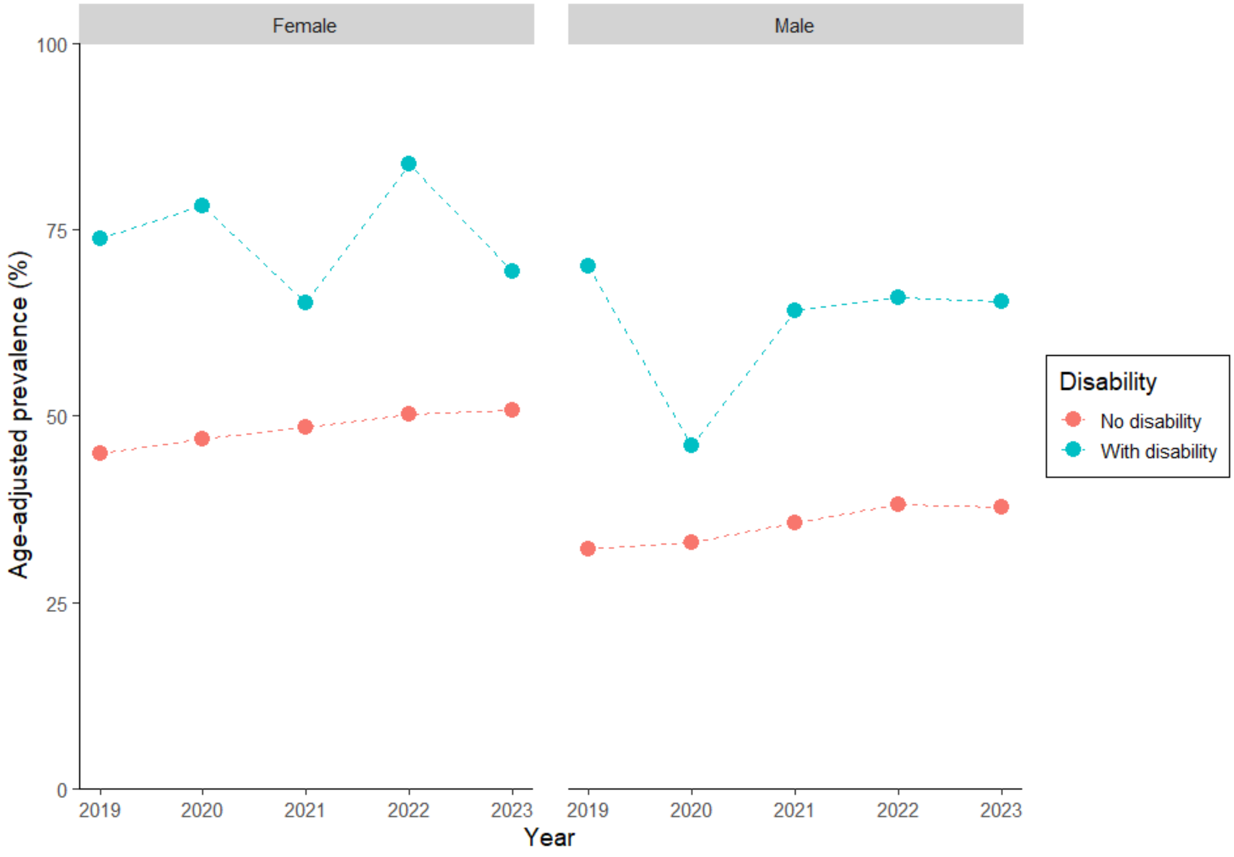

© 2026 Adzrago D et al. *JAMA Network Open*.

eFigure 29. Trends in Age-Standardized Anxiety/Depression Prevalence by Self-care Disability Status and Nativity Among Adults in the United States, 2019–2023

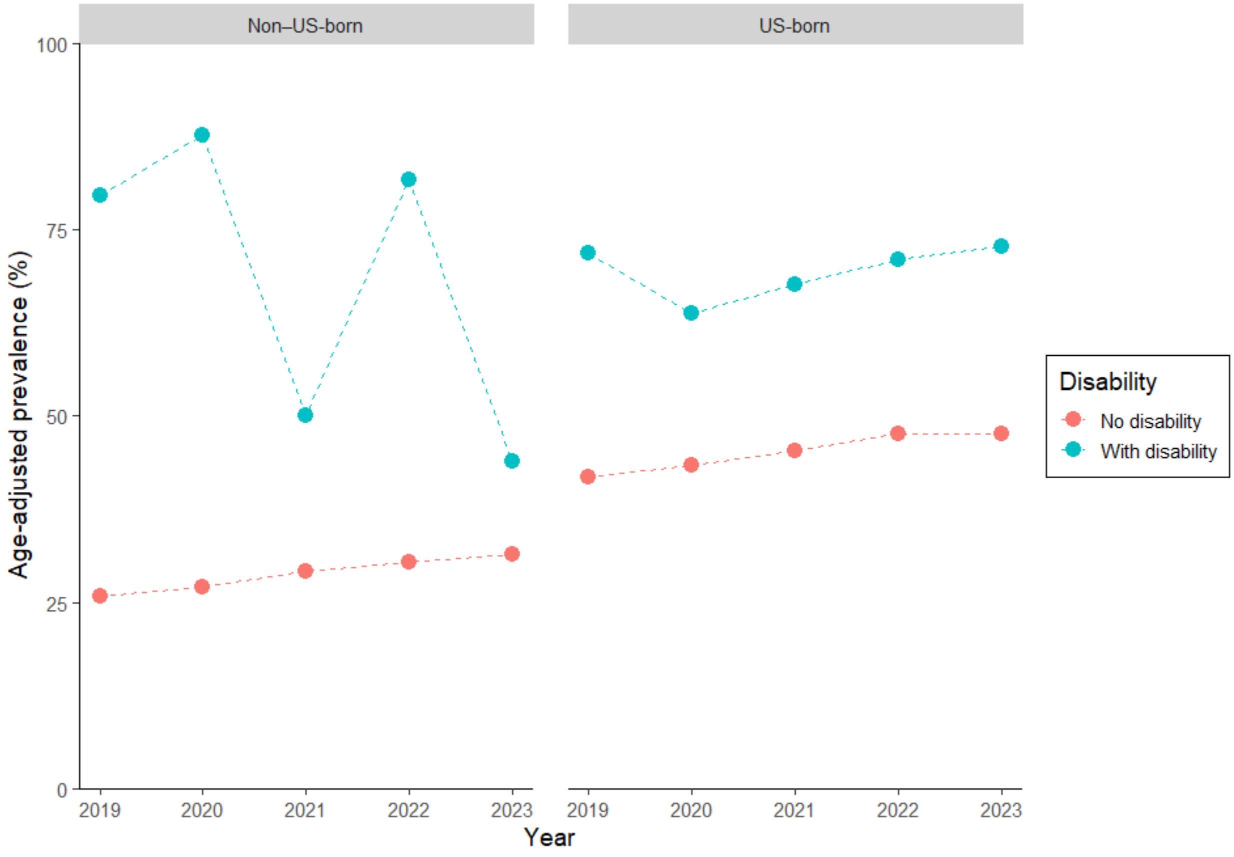

© 2026 Adzrago D et al. *JAMA Network Open*.

General Disability Status

eTable 1. Anxiety/Depression Prevalence by Disability Status and Survey Year Among Adults in the United States

	With no disability	With disability
	Anxiety/depression prevalence, % (95% CI)	
**Survey year**		
2019	36.58 (35.74-37.42)[a]	65.40 (62.12-68.69)[a]
2020	38.30 (37.43-39.18)[a]	67.02 (63.70-70.34)[a]
2021	40.53 (39.62-41.43)[a]	68.82 (65.92-71.73)[a]
2022	42.22 (41.34-43.10)[a]	72.06 (69.53-74.60)[a]
2023	42.42 (41.58-43.26)[a]	69.95 (67.29-72.61)[a]

Abbreviations: 95% CI, 95% confidence interval.

[a] Indicates statistical significance at $P < .01$.

eTable 2. Trends in Anxiety/Depression by Disability Status Among Adults in the United States, 2019–2023

Disability status	Range	AAPC, % (95% CI)
No disability	2019–2023	3.93 (2.15-5.75)[a]
With disability	2019–2023	2.08 (-0.49 to 4.72)

Abbreviations: AAPC, Average annual percentage change; 95% CI, 95% confidence interval.

[a] Indicates statistical significance at $P < .01$.

© 2026 Adzrago D et al. *JAMA Network Open*.

Disability Domains (Vision, Hearing, Mobility, Communication, Cognition, and Self-care)

Vision Disability

eTable 3. Trends in Anxiety/Depression by Vision Disability Status Among Adults in the United States, 2019–2023

Vision disability status	Range	AAPC, % (95% CI)
No disability	2019–2023	3.75 (2.09-5.44)[a]
With disability	2019–2023	1.90 (-5.79 to 10.23)

Abbreviations: AAPC, Average annual percentage change; 95% CI, 95% confidence interval.

[a] Indicates statistical significance at *P* < .01.

eTable 4. Trends in Anxiety/Depression by Race and/or Ethnicity and Vision Disability Status Among Adults in the United States, 2019–2023

Race and/or ethnicity and disability status	Range	AAPC, % (95% CI)
**Black**		
No disability	2019–2023	4.32 (0.35-8.44)[a]
With disability	2019–2023	3.31 (-11.21 to 20.20)
**Hispanic**		
No disability	2019–2023	3.73 (1.77-5.72)[b]
With disability	2019–2023	0.41 (-10.88 to 13.13)
**Other**		
No disability	2019–2023	5.77 (1.62-10.08)[a]
With disability	2019–2023	3.00 (-8.48 to 15.93)
**White**		
No disability	2019–2023	3.61 (1.91-5.33)[b]
With disability	2019–2023	0.40 (-15.69 to 19.57)

Abbreviations: AAPC, Average annual percentage change; 95% CI, 95% confidence interval.

[a] Indicates statistical significance at *P* < .05.

[b] Indicates statistical significance at *P* < .01.

© 2026 Adzrago D et al. *JAMA Network Open*.

eTable 5. Trends in Anxiety/Depression by Sex and Vision Disability Status Among Adults in the United States, 2019–2023

Sex and disability status	Range	AAPC, % (95% CI)
**Female**		
No disability	2019–2023	3.29 (2.19-4.40)[a]
With disability	2019–2023	-0.05 (-8.10 to 8.71)
**Male**		
No disability	2019–2023	4.57 (1.85-7.37)[b]
With disability	2019–2023	6.20 (-3.36 to 16.71)

Abbreviations: AAPC, Average annual percentage change; 95% CI, 95% confidence interval.

[a] Indicates statistical significance at *P* < .01.

[b] Indicates statistical significance at *P* < .05.

eTable 6. Trends in Anxiety/Depression by Nativity and Vision Disability Status Among Adults in the United States, 2019–2023

Nativity and disability status	Range	AAPC, % (95% CI)
**Non–US-born**		
No disability	2019–2023	5.19 (3.23-7.17)[a]
With disability	2019–2023	-1.73 (-13.90 to 12.16)
**US-born**		
No disability	2019–2023	3.51 (2.20-4.85)[a]
With disability	2019–2023	2.72 (-7.99 to 14.69)

Abbreviations: AAPC, Average annual percentage change; 95% CI, 95% confidence interval.

[a] Indicates statistical significance at *P* < .01.

© 2026 Adzrago D et al. *JAMA Network Open*.

Hearing Disability

eTable 7. Trends in Anxiety/Depression by Hearing Disability Status Among Adults in the United States, 2019–2023

Hearing disability status	Range	AAPC, % (95% CI)
No disability	2019–2023	3.74 (2.03-5.49)[a]
With disability	2019–2023	2.57 (-0.88 to 6.13)

Abbreviations: AAPC, Average annual percentage change; 95% CI, 95% confidence interval.

[a] Indicates statistical significance at *P* < .01.

eTable 8. Trends in Anxiety/Depression by Race and/or Ethnicity and Hearing Disability Status Among Adults in the United States, 2019–2023

Race and/or ethnicity and hearing disability status	Range	AAPC, % (95% CI)
**Black**		
No disability	2019–2023	4.35 (0.32-8.55)[a]
With disability	2019–2023	-6.15 (-19.91 to 9.97)
**Hispanic**		
No disability	2019–2023	3.88 (2.48-5.31)[b]
With disability	2019–2023	2.62 (-8.34 to 14.91)
**Other**		
No disability	2019–2023	5.69 (1.23-10.36)[a]
With disability	2019–2023	-2.60 (-34.50 to 44.82)
**White**		
No disability	2019–2023	3.50 (1.60-5.43)[b]
With disability	2019–2023	1.90 (-4.14 to 8.32)

Abbreviations: AAPC, Average annual percentage change; 95% CI, 95% confidence interval.

[a] Indicates statistical significance at *P* < .05.

[b] Indicates statistical significance at *P* < .01.

eTable 9. Trends in Anxiety/Depression by Sex and Hearing Disability Status Among Adults in the United States, 2019–2023

Sex and hearing disability status	Range	AAPC, % (95% CI)
**Female**		
No disability	2019–2023	3.21 (1.95-4.48)[a]
With disability	2019–2023	2.86 (-1.82 to 7.76)
**Male**		
No disability	2019–2023	4.68 (1.89-7.55)[b]
With disability	2019–2023	4.74 (-8.07 to 19.33)

Abbreviations: AAPC, Average annual percentage change; 95% CI, 95% confidence interval.

[a] Indicates statistical significance at *P* < .01.

[b] Indicates statistical significance at *P* < .05.

© 2026 Adzrago D et al. *JAMA Network Open*.

eTable 10. Trends in Anxiety/Depression by Nativity and Hearing Disability Status Among Adults in the United States, 2019–2023

Nativity and hearing disability status	Range	AAPC, % (95% CI)
**Non–US-born**		
No disability	2019–2023	5.24 (3.43-7.08)[a]
With disability	2019–2023	5.04 (-7.42 to 19.18)
**US-born**		
No disability	2019–2023	3.47 (2.02-4.93)[a]
With disability	2019–2023	1.47 (-1.03 to 4.03)

Abbreviations: AAPC, Average annual percentage change; 95% CI, 95% confidence interval.

[a] Indicates statistical significance at $P < .01$.

© 2026 Adzrago D et al. *JAMA Network Open*.

Mobility Disability

eTable 11. Trends in Anxiety/Depression by Mobility Disability Status Among Adults in the United States, 2019–2023

Mobility disability status	Range	AAPC, % (95% CI)
No disability	2019–2023	4.03 (2.41-5.68)[a]
With disability	2019–2023	2.43 (-4.91 to 10.33)

Abbreviations: AAPC, Average annual percentage change; 95% CI, 95% confidence interval.

[a] Indicates statistical significance at *P* < .01.

eTable 12. Trends in Anxiety/Depression by Race and/or Ethnicity and Mobility Disability Status Among Adults in the United States, 2019–2023

Race and/or ethnicity and mobility disability status	Range	AAPC, % (95% CI)
**Black**		
No disability	2019–2023	4.74 (0.90-8.71)[a]
With disability	2019–2023	7.78 (-8.65 to 27.17)
**Hispanic**		
No disability	2019–2023	3.87 (2.18-5.58)[b]
With disability	2019–2023	5.20 (-1.43 to 12.28)
**Other**		
No disability	2019–2023	6.24 (1.37-11.35)[a]
With disability	2019–2023	7.56 (-14.43 to 35.19)
**White**		
No disability	2019–2023	3.84 (1.95-5.76)[b]
With disability	2019–2023	0.01 (-4.83 to 5.09)

Abbreviations: AAPC, Average annual percentage change; 95% CI, 95% confidence interval.

[a] Indicates statistical significance at *P* < .05.

[b] Indicates statistical significance at *P* < .01.

eTable 13. Trends in Anxiety/Depression by Sex and Mobility Disability Status Among Adults in the United States, 2019–2023

Sex and mobility disability status	Range	AAPC, % (95% CI)
**Female**		
No disability	2019–2023	3.45 (2.23-4.69)[a]
With disability	2019–2023	3.16 (-3.38 to 10.14)
**Male**		
No disability	2019–2023	4.94 (2.25-7.70)[a]
With disability	2019–2023	1.17 (-7.39 to 10.52)

Abbreviations: AAPC, Average annual percentage change; 95% CI, 95% confidence interval.

[a] Indicates statistical significance at *P* < .01.

© 2026 Adzrago D et al. *JAMA Network Open*.

eTable 14. Trends in Anxiety/Depression by Nativity and Mobility Disability Status Among Adults in the United States, 2019–2023

Nativity and mobility disability status	Range	AAPC, % (95% CI)
**Non–US-born**		
No disability	2019–2023	5.52 (3.74-7.33)[a]
With disability	2019–2023	-5.51 (-20.66 to 12.53)
**US-born**		
No disability	2019–2023	3.74 (2.22-5.28)[a]
With disability	2019–2023	2.61 (-4.95 to 10.78)

Abbreviations: AAPC, Average annual percentage change; 95% CI, 95% confidence interval.

[a] Indicates statistical significance at $P < .01$.

© 2026 Adzrago D et al. *JAMA Network Open*.

Communication Disability

eTable 15. Trends in Anxiety/Depression by Communication Disability Status Among Adults in the United States, 2019–2023

Communication disability status	Range	AAPC, % (95% CI)
No disability	2019–2023	3.76 (1.98-5.58)[a]
With disability	2019–2023	-1.39 (-6.91 to 4.45)

Abbreviations: AAPC, Average annual percentage change; 95% CI, 95% confidence interval.

[a] Indicates statistical significance at *P* < .01.

eTable 16. Trends in Anxiety/Depression by Race and/or Ethnicity and Communication Disability Status Among Adults in the United States, 2019–2023

Race and/or ethnicity and communication disability status	Range	AAPC, % (95% CI)
**Black**		
No disability	2019–2023	4.42 (0.40-8.60)[a]
With disability	2019–2023	-10.15 (-30.76 to 16.60)
**Hispanic**		
No disability	2019–2023	3.70 (1.86-5.58)[b]
With disability	2019–2023	7.96 (-5.89 to 23.85)
**Other**		
No disability	2019–2023	5.85 (1.08-10.84)[a]
With disability	2019–2023	-2.44 (-29.02 to 34.09)
**White**		
No disability	2019–2023	3.60 (1.84-5.38)[b]
With disability	2019–2023	-1.58 (-11.13 to 8.98)

Abbreviations: AAPC, Average annual percentage change; 95% CI, 95% confidence interval.

[a] Indicates statistical significance at *P* < .05.

[b] Indicates statistical significance at *P* < .01.

eTable 17. Trends in Anxiety/Depression by Sex and Communication Disability Status Among Adults in the United States, 2019–2023

Sex and communication disability status	Range	AAPC, % (95% CI)
**Female**		
No disability	2019–2023	3.24 (2.05-4.44)[a]
With disability	2019–2023	0.73 (-13.90 to 17.84)
**Male**		
No disability	2019–2023	4.64 (1.72-7.65)[b]
With disability	2019–2023	-3.57 (-5.98 to -1.10)[b]

Abbreviations: AAPC, Average annual percentage change; 95% CI, 95% confidence interval.

[a] Indicates statistical significance at *P* < .01.

[b] Indicates statistical significance at *P* < .05.

© 2026 Adzrago D et al. *JAMA Network Open*.

eTable 18. Trends in Anxiety/Depression by Nativity and Communication Disability Status Among Adults in the United States, 2019–2023

Nativity and communication disability status	Range	AAPC, % (95% CI)
**Non–US-born**		
No disability	2019–2023	5.05 (3.46-6.67)[a]
With disability	2019–2023	3.56 (-23.71 to 40.58)
**US-born**		
No disability	2019–2023	3.53 (2.00-5.10)[a]
With disability	2019–2023	-2.46 (-7.15 to 2.46)

Abbreviations: AAPC, Average annual percentage change; 95% CI, 95% confidence interval.

[a] Indicates statistical significance at $P < .01$.

© 2026 Adzrago D et al. *JAMA Network Open*.

Cognition Disability

eTable 19. Trends in Anxiety/Depression by Cognition Disability Status Among Adults in the United States, 2019–2023

Cognition disability status	Range	AAPC, % (95% CI)
No disability	2019–2023	3.76 (2.00-5.55)[a]
With disability	2019–2023	1.33 (-0.97 to 3.69)

Abbreviations: AAPC, Average annual percentage change; 95% CI, 95% confidence interval.

[a] Indicates statistical significance at *P* < .01.

eTable 20. Trends in Anxiety/Depression by Race and/or Ethnicity and Cognition Disability Status Among Adults in the United States, 2019–2023

Race and/or ethnicity and cognition disability status	Range	AAPC, % (95% CI)
**Black**		
No disability	2019–2023	4.11 (-0.01 to 8.41)
With disability	2019–2023	0.54 (-3.86 to 5.14)
**Hispanic**		
No disability	2019–2023	3.68 (1.86-5.52)[a]
With disability	2019–2023	1.75 (-7.47 to 11.90)
**Other**		
No disability	2019–2023	6.67 (1.44-12.18)[b]
With disability	2019–2023	-1.76 (-10.91 to 8.33)
**White**		
No disability	2019–2023	3.48 (1.79-5.21)[a]
With disability	2019–2023	1.25 (-1.98 to 4.59)

Abbreviations: AAPC, Average annual percentage change; 95% CI, 95% confidence interval.

[a] Indicates statistical significance at *P* < .01.

[b] Indicates statistical significance at *P* < .05.

eTable 21. Trends in Anxiety/Depression by Sex and Cognition Disability Status Among Adults in the United States, 2019–2023

Sex and cognition disability status	Range	AAPC, % (95% CI)
**Female**		
No disability	2019–2023	3.21 (2.09-4.35)[a]
With disability	2019–2023	0.82 (-3.59 to 5.44)
**Male**		
No disability	2019–2023	4.59 (1.58-7.69)[b]
With disability	2019–2023	1.79 (-1.56 to 5.25)

Abbreviations: AAPC, Average annual percentage change; 95% CI, 95% confidence interval.

[a] Indicates statistical significance at *P* < .01.

[b] Indicates statistical significance at *P* < .05.

© 2026 Adzrago D et al. *JAMA Network Open*.

eTable 22. Trends in Anxiety/Depression by Nativity and Cognition Disability Status Among Adults in the United States, 2019–2023

Nativity and cognition disability status	Range	AAPC, % (95% CI)
**Non–US-born**		
No disability	2019–2023	5.16 (2.50-7.88)[a]
With disability	2019–2023	1.45 (-6.79 to 10.42)
**US-born**		
No disability	2019–2023	3.52 (2.11-4.95)[a]
With disability	2019–2023	1.19 (-1.26 to 3.70)

Abbreviations: AAPC, Average annual percentage change; 95% CI, 95% confidence interval.

[a] Indicates statistical significance at $P < .01$.

© 2026 Adzrago D et al. *JAMA Network Open*.

Self-care Disability

eTable 23. Trends in Anxiety/Depression by Self-care Disability Status Among Adults in the United States, 2019–2023

Self-care disability status	Range	AAPC, % (95% CI)
No disability	2019–2023	3.75 (2.06-5.46)[a]
With disability	2019–2023	-0.05 (-6.03 to 6.32)

Abbreviations: AAPC, Average annual percentage change; 95% CI, 95% confidence interval.

[a] Indicates statistical significance at *P* < .01.

eTable 24. Trends in Anxiety/Depression by Race and/or Ethnicity and Self-care Disability Status Among Adults in the United States, 2019–2023

Race and/or ethnicity and self-care disability status	Range	AAPC, % (95% CI)
**Black**		
No disability	2019–2023	4.22 (0.11-8.49)[a]
With disability	2019–2023	0.34 (-16.65 to 20.79)
**Hispanic**		
No disability	2019–2023	3.56 (1.88-5.28)[b]
With disability	2019–2023	7.08 (-3.36 to 18.64)
**Other**		
No disability	2019–2023	5.82 (1.20-10.65)[a]
With disability	2019–2023	-10.52 (-22.14 to 2.83)
**White**		
No disability	2019–2023	3.56 (1.67-5.48)[b]
With disability	2019–2023	0.09 (-4.01 to 4.38)

Abbreviations: AAPC, Average annual percentage change; 95% CI, 95% confidence interval.

[a] Indicates statistical significance at *P* < .05.

[b] Indicates statistical significance at *P* < .01.

eTable 25. Trends in Anxiety/Depression by Sex and Self-care Disability Status Among Adults in the United States, 2019–2023

Sex and self-care disability status	Range	AAPC, % (95% CI)
**Female**		
No disability	2019–2023	3.22 (2.19-4.27)[a]
With disability	2019–2023	0.19 (-10.42 to 12.05)
**Male**		
No disability	2019–2023	4.66 (1.86-7.54)[b]
With disability	2019–2023	0.19 (-12.63 to 14.89)

Abbreviations: AAPC, Average annual percentage change; 95% CI, 95% confidence interval.

[a] Indicates statistical significance at *P* < .01.

[b] Indicates statistical significance at *P* < .05.

© 2026 Adzrago D et al. *JAMA Network Open*.

eTable 26. Trends in Anxiety/Depression by Nativity and Self-care Disability Status Among Adults in the United States, 2019–2023

Nativity and self-care disability status	Range	AAPC, % (95% CI)
**Non–US-born**		
No disability	2019–2023	5.20 (3.73-6.70)[a]
With disability	2019–2023	-11.54 (-30.68 to 12.88)
**US-born**		
No disability	2019–2023	3.51 (2.10-4.95)[a]
With disability	2019–2023	1.09 (-3.45 to 5.85)

Abbreviations: AAPC, Average annual percentage change; 95% CI, 95% confidence interval.

[a] Indicates statistical significance at $P < .01$.

© 2026 Adzrago D et al. *JAMA Network Open*.
