# Supplementary material for: Anxiety or Depression Trends by Disability Status and Demographic Intersections in US Adults, 2019-2023
Source: JAMA Netw Open. 2026 Feb 2;9(2):e2557332. doi: 10.1001/jamanetworkopen.2025.57332 (PMC12865658; doi:10.1001/jamanetworkopen.2025.57332)
Supplement: Supplement 2. — Data Sharing Statement [file jamanetwopen-e2557332-s002.pdf]

Data Sharing Statement

Adzrago. Anxiety or Depression Trends by Disability Status and Demographic Intersections in US Adults, 2019-2023. *JAMA Netw Open*. Published February 02, 2026. doi:10.1001/jamanetworkopen.2025.57332

Data
Data available: Yes
Data types: Data (not involving human participants)
How to access data: All data in this study are de-identified and publicly available at https://www.cdc.gov/nchs/nhis/
When available: With publication

Supporting Documents
Document types: None

Additional Information
Who can access the data: All data in this study are de-identified and publicly available at https://www.cdc.gov/nchs/nhis/
Types of analyses: All data in this study are de-identified and publicly available at https://www.cdc.gov/nchs/nhis/
Mechanisms of data availability: All data in this study are de-identified and publicly available at https://www.cdc.gov/nchs/nhis/
